# Supplementary material for: Anti-cancer Effects of a Chemically Modified miR-143 on Bladder Cancer by Either Systemic or Intravesical Treatment
Source: Mol Ther Methods Clin Dev. 2019 Feb 20;13:290–302. doi: 10.1016/j.omtm.2019.02.005 (PMC6416526; doi:10.1016/j.omtm.2019.02.005)
Supplement: Document S1. Supplemental Materials and Methods and Figures S1–S4 [file mmc1.pdf]

OMTM, Volume *13*

Supplemental Information

Anti-cancer Effects of a Chemically Modified miR-143 on Bladder Cancer by Either Systemic or Intravesical Treatment

Yuki Yoshikawa, Kohei Taniguchi, Takuya Tsujino, Kazuki Heishima, Teruo Inamoto, Tomoaki Takai, Koichiro Minami, Haruhito Azuma, Kanjiro Miyata, Kotaro Hayashi, Kazunori Kataoka, and Yukihiro Akao

Materials and methods

Clinical data analysis

Clinical sample data was quoted from available database c-Bioportal for cancer genomics (http://www.cbioportal.org/). Clinical samples included from the combination of 9 cohorts are 1412 specimens. K-RAS, H-RAS and N-RAS mutation in the sample were analyzed.

RNA degradation

In order to confirm the resistance of chemically modified miR-143 to RNAse, RNA degradation experiment was carried out. Compared Ambion's miR-143 and chemically modified miR-143s. MiR-143s are administered to 10% FBS solution media and RNA equilibrium is established by injecting PCI solution in 1, 2, 3, 4, 5, 10, 20 and 30 minutes. RNA was extracted from the solution and the amount of miR143 was measured by real time PCR. The ratio of phenol, chloroform, and isoamyl alcohol are 25: 24: 1.

Patients and their samples

All human samples were obtained from patients who had undergone biopsy or surgery for resection at Osaka Medical College Hospital (Takatsuki, Osaka, Japan). Informed consent in writing was obtained from each patient. This is exactly the same as the sample used in Fig. 1.

Transfection experiments

253J-BV cells were seeded into 6-well plates at a concentration of 0.5 × 10^5 per well (10-30% confluence) on the day before the transfection. siRNAs were used: In treatment experiments, the sequence of the siR-SOS1, 5'-CGG CAU GUA CUA CAG GCC UGU UUA-3'. The effects manifested by the introduction of siR-SOS1 into the cells were assessed at 72 h after the transfection as mRNA expression level, protein levels of RAS and RAS related genes, and cell proliferation. We used the same dose of Lipofectamine™ RNAiMAX in all transfection experiments.

Real-time reverse transcription PCR

The detail methods of isolation of RNA and RT-PCR were described in main document. The primers for *SOS1* and *GAPDH* were the following: *SOS1*-sense, 5'-GGA GGA GTG TCC CAA TTT ATT AG-3', and *SOS1*-antisense, 5'-TTT CAT TGG CTC ATG TAT AAG GG-3'; *GAPDH*-sense, 5'-CCA CCC ATG GCA AAT TCC ATG GCA-3', and *GAPDH*-antisense, 5'-TCT AGA CGG CAG GTC AGG TCC ACC-3'.*GAPDH* was used as internal controls. The relative expression levels were calculated by use of the ΔΔCt method.

Western blot analysis

Protein extraction and Western blotting analysis were performed as described in main document. The following primary antibodies were used:

antibodies against, c-Myc, p-AKT, AKT, p-ERK, ERK, SOS1, and GAPDH (Cell Signaling Technology, Inc., Danvers, MA, USA);, Total RAS (Abcam, Cambridge, UK);, and K-RAS, H-RAS (Santa Cruz Biotechnology). Anti-rat, anti-rabbit, and horse anti-mouse IgG (Cell Signaling Technology) were used as secondary antibodies. GAPDH served as an internal control.

In vivo orthotopic model

The method of transplantation of the tumor and time table of administering the medicine were carried out as described in the main document. MiR-143 was administered in 2 ways except the control miRNA. One way was that Syn-miR-143s (#12; 83 μg/kg per 1 administration) complexed with 10 μl of HEPES (1mM), 10 μl of Opti-MEM that had been incubated with 1 μl of Lipofectamine^{TM} RNAiMAX (Invitrogen), and 80 μl of saline were delivered intravesically every other day. For the other administration, Syn-miR-143s (#12; 83 μg/kg per 1 administration) was mixed with the block copolymer to formulate the PIC nanocarriers as described above and then delivered 8 times intravesically every other day. After the administration, some mice were sacrificed at around 30 days after the inoculation of the cells. RNA was extracted from several organs. Some organs were stained with hematoxylin and eosin (H-E).

Supplementary Figure.1

A

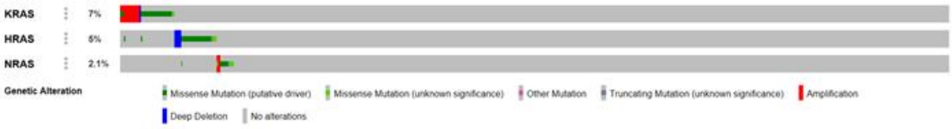

Supplementary Figure.2

A

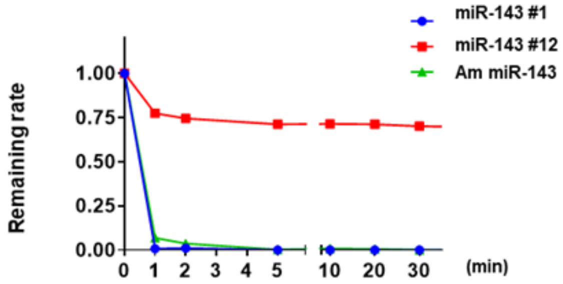

Supplementary Figure.3

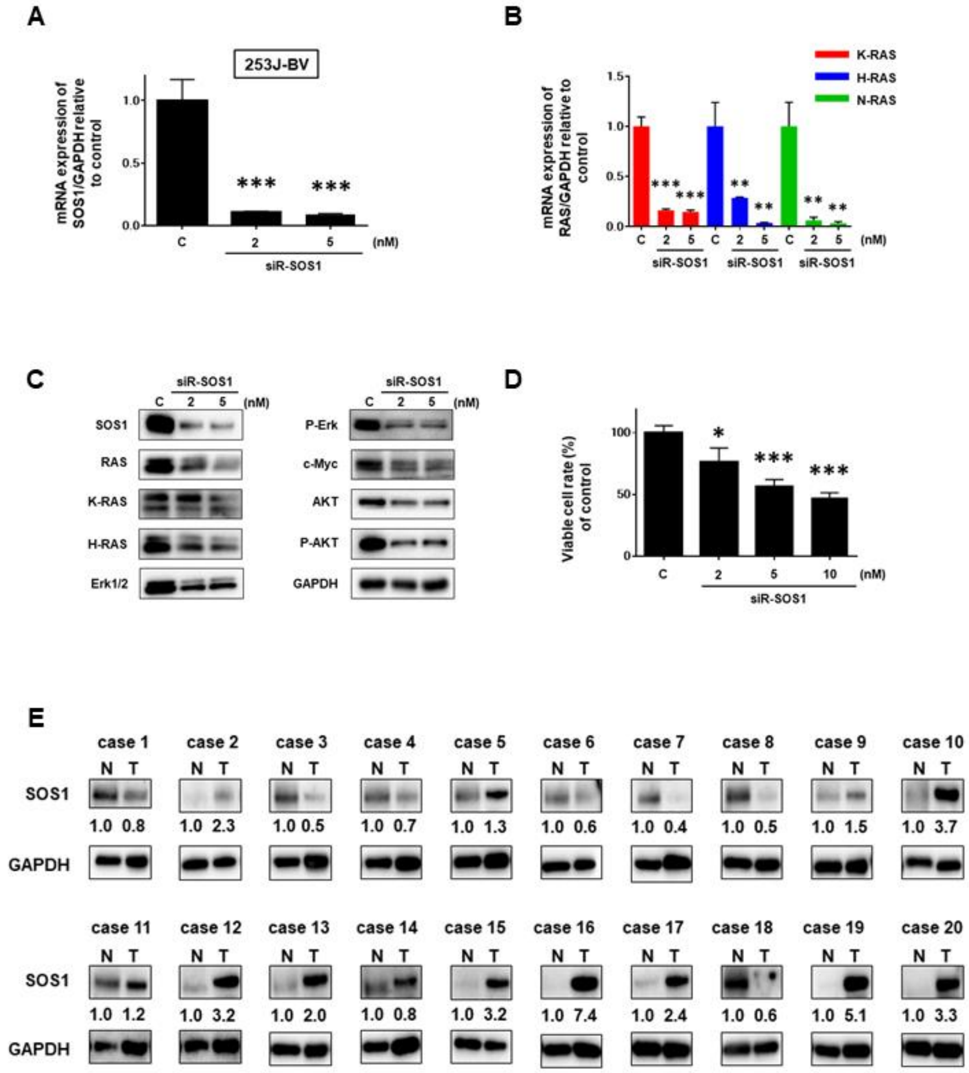

Supplementary Figure.4

A

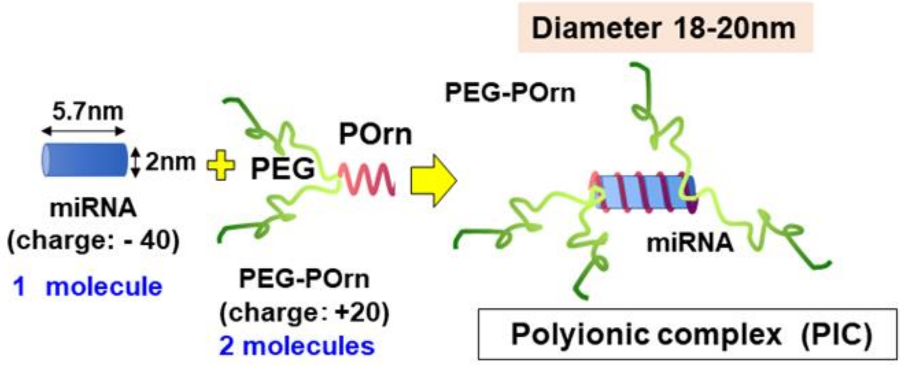

B

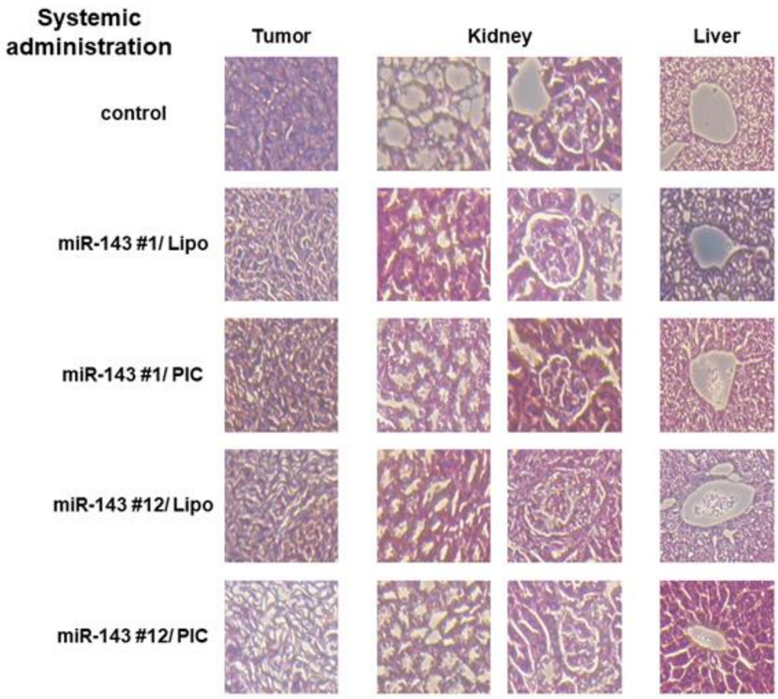

C

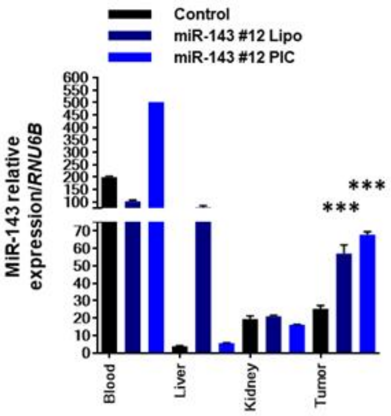

D

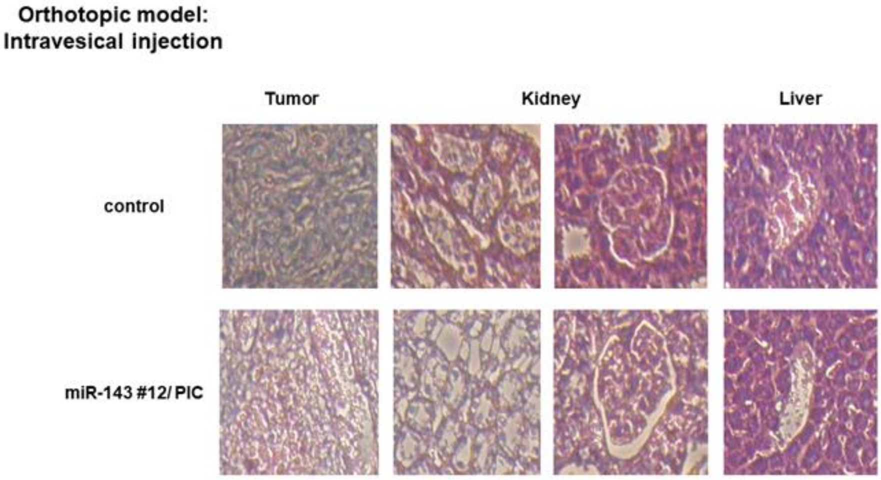
Orthotopic model:
Intravesical injection
Tumor
Kidney
Liver
control
miR-143 #12/ PIC

Supplemental Figures

Supplementary Figure 1. Mutation of RAS in clinical samples of bladder cancer.

A. Clinical significance of RAS mutation in BC. Patients are in 9 studies from available datasets. cBioportal for cancer genomics (http://www.cbioportal.org/) was used.

Supplementary Figure 2. Stability of miR-143s used in this study

A. Decay of miR-143 after the incubation of miR-143s in 5%FCS solution.

Supplementary Figure 3. Role of SOS1 in regulating RAS gene in BC cells.

A. mRNA expression of SOS1 after transfection with siR-SOS1 (2 nM, 5 nM).

B. mRNA expression of RAS isomer (K-RAS, H-RAS, and N-RAS) at 72 h after transfection with siR-SOS1 (2 nM, 5 nM).

C. Levels of RAS and RAS-related proteins at 72 h after transfection with siR-SOS1 (2 nM, 5 nM).

D. Cell viability at 72 h after transfection with siR-SOS1 (2 nM, 5 nM, 10 nM).

E. SOS1 expression in 20 clinical bladder cancer samples as determined

by Western blot analysis.

Supplementary Figure 4. Formulation of PIC and histopathological findings for tumor, kidney, and liver after treatments of 253J-BV cell-xenografted mice

A. Formulation of each miR-143/PIC. POrn: poly(Ornithine)

B. Histopathology of tumor/kidney/liver after treatment of subcutaneous xenograft mice with miR-143#1(Lipo or PIC) or miR-143#12(Lipo or PIC).

C. Tissue distribution of miR-143 in the miR-143#12/Lipo and miR-143#12/PIC groups (Blood/Tumor/ Kidney/ Liver).

D. Histopathology of tumor/kidney/liver after intravesical infusion with miR-143#12/PIC in orthotopic xenograft model.
