# Supplementary material for: Anti-cancer Effects of a Chemically Modified miR-143 on Bladder Cancer by Either Systemic or Intravesical Treatment
Source: Mol Ther Methods Clin Dev. 2019 Feb 20;13:290–302. doi: 10.1016/j.omtm.2019.02.005 (PMC6416526; doi:10.1016/j.omtm.2019.02.005)
Supplement: Document S2. Article plus Supplemental Information [file mmc2.pdf]

Molecular Therapy
Methods & Clinical Development
Original Article

AMERICAN SOCIETY of GENE & CELL THERAPY

Anti-cancer Effects of a Chemically Modified miR-143 on Bladder Cancer by Either Systemic or Intravesical Treatment

Yuki Yoshikawa,[1,2] Kohei Taniguchi,[3] Takuya Tsujino,[1,2] Kazuki Heishima,[1] Teruo Inamoto,[2] Tomoaki Takai,[1,2] Koichiro Minami,[1,2] Haruhito Azuma,[2] Kanjiro Miyata,[4] Kotaro Hayashi,[5] Kazunori Kataoka,[5,6] and Yukihiro Akao[1]

[1]United Graduate School of Drug Discovery and Medical Information Sciences, Gifu University, 1-1 Yanagido, Gifu 501-1193, Japan; [2]Department of Urology, Osaka Medical College, 2-7 Daigaku-machi, Takatsuki, Osaka 569-8686, Japan; [3]Translational Research Program, Osaka Medical College, 2-7 Daigaku-machi, Takatsuki, Osaka 569-8686, Japan; [4]Department of Materials Engineering, Graduate School of Engineering, The University of Tokyo, 7-3-1 Hongo, Bunkyo-ku, Tokyo 113-8656, Japan; [5]Inovation Center of NanoMedicine, Kawasaki Institute of Industrial Promotion, 3-25-14 Tonomachi, Kawasaki-ku, Kawasaki 210-0821, Japan; [6]Policy Alternatives Research Institute, The University of Tokyo, 7-3-1 Hongo, Bunkyo-ku, Tokyo 113-0033, Japan

We developed a novel chemically modified miR-143 (miR-143#12), and with it we investigated the contribution of miR-143 to the pathogenesis of bladder cancer (BC), in which miR-143 is extremely downregulated. Since miR-143 silenced K-RAS and RAS effector-signaling molecules Erk and Akt, we performed the ectopic expression of miR-143 in human BC 253J-BV cells, and we examined the growth inhibition and the mechanism of it *in vitro* and in orthotopic model mice. As a result, miR-143#12 induced a marked growth inhibition with apoptosis through impairing RAS-signaling networks, including SOS1, which exchanges guanosine diphosphate (GDP)/RAS for active guanosine triphosphate (GTP)/RAS. In the *in vivo* study, miR-143#12 exhibited a marked anti-tumor activity by either systemic or intravesical administration with polyionic copolymer (PIC) as the carrier, compared with the activity obtained by use of lipofection. These findings raised the possibility that the chemically modified miR-143#12 would be a candidate of microRNA (miRNA) medicine for BC delivered by intravesical infusion.

INTRODUCTION

Bladder cancer (BC) is one of the most common cancers of the urogenital system. Over 20,000 new cases of BC were identified in Japan in 2015, and approximately over 8,000 deaths from BC were anticipated. BC can be classified into 2 types, i.e., muscle-invasive BC (MIBC) and non-muscle-invasive BC (NMIBC). NMIBC can be managed with transurethral resection of the bladder tumor and intravesical chemotherapy and/or immunotherapy.[1] Compared with NMIBC, MIBC is a highly aggressive disease. For patients with NMIBC, the issue is to prevent tumor recurrence, which occurs in 50%–90% of the patients within 5 years, and, most importantly, disease progression to muscle invasion, which occurs in up to 20% of patients.[2] In particular, carcinoma *in situ* (CIS) is regarded as a problem. It is classified as NMIBC, but its recurrence rate is as high as 90% and treatment for it, bladder preservation, is ineffective. On the other hand, patients who are diagnosed as having MIBC also have an unfavorable prognosis, with a 5-year overall and cancer-specific survival period estimated to be approximately 60% because of no effective drug.[3,4] Thus, there is a need to identify the driver genes and to develop a more effective therapeutic strategy for BC.

MicroRNAs (miRNAs) are endogenous small non-coding RNA molecules (19–22 nt in length) that regulate protein-coding gene expression by binding to the 3′ UTR of mRNAs. Increasing evidence suggests that miRNAs are aberrantly expressed in various human cancers and that they play significant roles in cancer initiation, development, and metastasis.[5–7] miRNAs potently influence cellular activities through the regulation of extensive gene expression networks. Our team has been focusing on the studies for the development of RNA medicine targeting plural genes through RNAi by the replacement of tumor suppressor (TS)-miRNAs. miR-143 is one of the representative TS-miRNAs that is poorly expressed in a variety of cancers, including BC.[8–10] miR-143 has been shown to act as a tumor suppressor in non-small-cell lung cancer,[11] cervical cancer,[12] prostate cancer,[13] ovarian cancer,[14] colon cancer,[15] and leukemia[16,17] and to silence not only K-RAS[18] but also RAS-effector signal genes Erk and Akt.[19] So far, we have been exploring the development of RNA medicine of miR-143 for RAS-driven cancers, because miR-143 perturbs K-RAS-signaling networks systematically.[20]

The RAS gene, which is expressed as 3 isoforms, K-RAS, H-RAS, and N-RAS, is one of the most well-known oncogenes,[21] and the frequency of mutations of both K-RAS and H-RAS has been reported to be almost 10% in BC.[22] The contribution of the RAS gene to the

Received 7 January 2019; accepted 13 February 2019;
https://doi.org/10.1016/j.omtm.2019.02.005.

Correspondence: Yukihiro Akao, MD, United Graduate School of Drug Discovery and Medical Information Sciences, Gifu University, 1-1 Yanagido, Gifu 501-1193, Japan.
E-mail: yakao@gifu-u.ac.jp

 © 2019 The Authors.
This is an open access article under the CC BY license (http://creativecommons.org/licenses/by/4.0/).

Table.1. Clinicopathological Patient Features

Case	Age	Sex	Size (cm)	Grade	T Stage	CIS	miR-143	RAS	Meta
1	81	M	2	3	pTa	no	D	U	(−)
2	75	M	2	3	pT1	no	D	U	(−)
3	73	M	2	3	pT1	no	D	U	(−)
4	60	F	2	1	pT2	no	D	U	(−)
5	80	M	2	3	pT1	no	D	U	(−)
6	83	M	1.5	2	pT2	no	D	U	(−)
7	76	M	2.5	2	pT1	yes	D	U	(−)
8	61	M	2.5	3	pT2	no	D	U	(−)
9	48	F	5	3	pT2	no	D	U	(−)
10	52	M	3	3	pT2	yes	D	U	(−)
11	75	M	1.5	1	pTa	no	D	U	(−)
12	79	F	1.5	3	pTa	yes	D	U	(−)
13	60	M	1	1	pTa	fair	D	U	(−)
14	86	M	2	2	pT1	fair	D	U	(−)
15	69	M	1	1	pTa	fair	D	U	(−)
16	66	M	3	3	pT2	yes	D	U	(−)
17	77	M	1	1	pTa	no	D	U	(−)
18	63	M	5	2	pTa	no	D	U	(−)
19	68	M	1	1	pTa	no	D	U	(−)
20	79	F	2	2	pT1	no	D	U	(−)

M, male; F, female; U, upregulation RAS expression level; D, downregulation of miR-143 relative ratio; grade 1, well-differentiated type carcinoma; grade 2, moderately differentiated type carcinoma; grade 3, poorly differentiated type carcinoma; pTa, papiloma non-invasive carcinoma; pT1, non-muscle-invasive carcinoma; pT2, muscle-invasive carcinoma; CIS, carcinoma *in situ*; Meta, metastasis.

pathogenesis of BC has already been reported,[21,23] but the detailed mechanism has not been elucidated.

In this study, we examined the expression levels of RAS and miR-143 in human BC clinical samples, including some CISs, and we clarified the correlation between them. For the development of RNA medicine against RAS-driven cancers, we produced more than 100 chemically modified miR-143 derivatives. Among them, we found an RNase-resistant and potent miR-143 that was chemically modified only in the guide strand. By using this miR-143, we were able to unveil the networks of RAS-signaling pathways and the oncogenic roles of K-RAS and H-RAS in BC cells, and we showed the possibility that the novel synthetic miR-143 would be applied to early BC by intravesical infusion.

RESULTS

Expression of miR-143 Was Extremely Downregulated in Clinical Tumor Samples from BC Patients

We first examined the expression levels of miR-143 in BC tumor and adjacent normal tissue samples from the same patient (Table 1). Totally, 20 cases were examined. The expression levels of miR-143 in the clinical tumor samples examined by real-time PCR were extremely downregulated compared with those in the adjacent normal tissues (Figure 1A). Since miR-143 silences K-RAS,[24] the expression levels of total RAS, K-RAS, and H-RAS were evaluated by performing western blot analysis of the same clinical samples. In all cases tested, K-RAS and H-RAS protein expression levels were significantly increased in the BC tumors compared with those in the normal tissue samples (Figure 1B). Importantly, both K-RAS and H-RAS were upregulated in the tumor samples, and an inverse correlation was found between miR-143 and K-RAS or H-RAS (Figure 1C). On the other hand, a positive relation was found between H-RAS and K-RAS (Figure 1C). Also, dataset analysis of 1,314 samples of various BC showed that K-RAS mutation was in 86 cases (7%), H-RAS mutation was in 71 cases (5%), and N-RAS mutation was in 28 cases (2.1%) (Figure S1A). These findings suggested that not only H-RAS but also K-RAS might play pivotal roles in the pathogenesis of BC and that their overexpression in the tumor samples was closely associated with the downregulation of miR-143.

The Expression Levels of K-RAS and H-RAS Were Upregulated in miR-143-Downregulated Human BC 253J-BV Cells

We examined the levels of miR-143 and RAS isoforms in human normal transitional human urothelial cells (HUCs) and in the BC 253J-BV cell line used in this study. As shown in Figure 1D, K-RAS was the major isomer (approximately 70%) as judged from the mRNA levels in 253J-BV cells. On the other hand, the expression level of miR-143 in 253J-BV cells was extremely downregulated compared

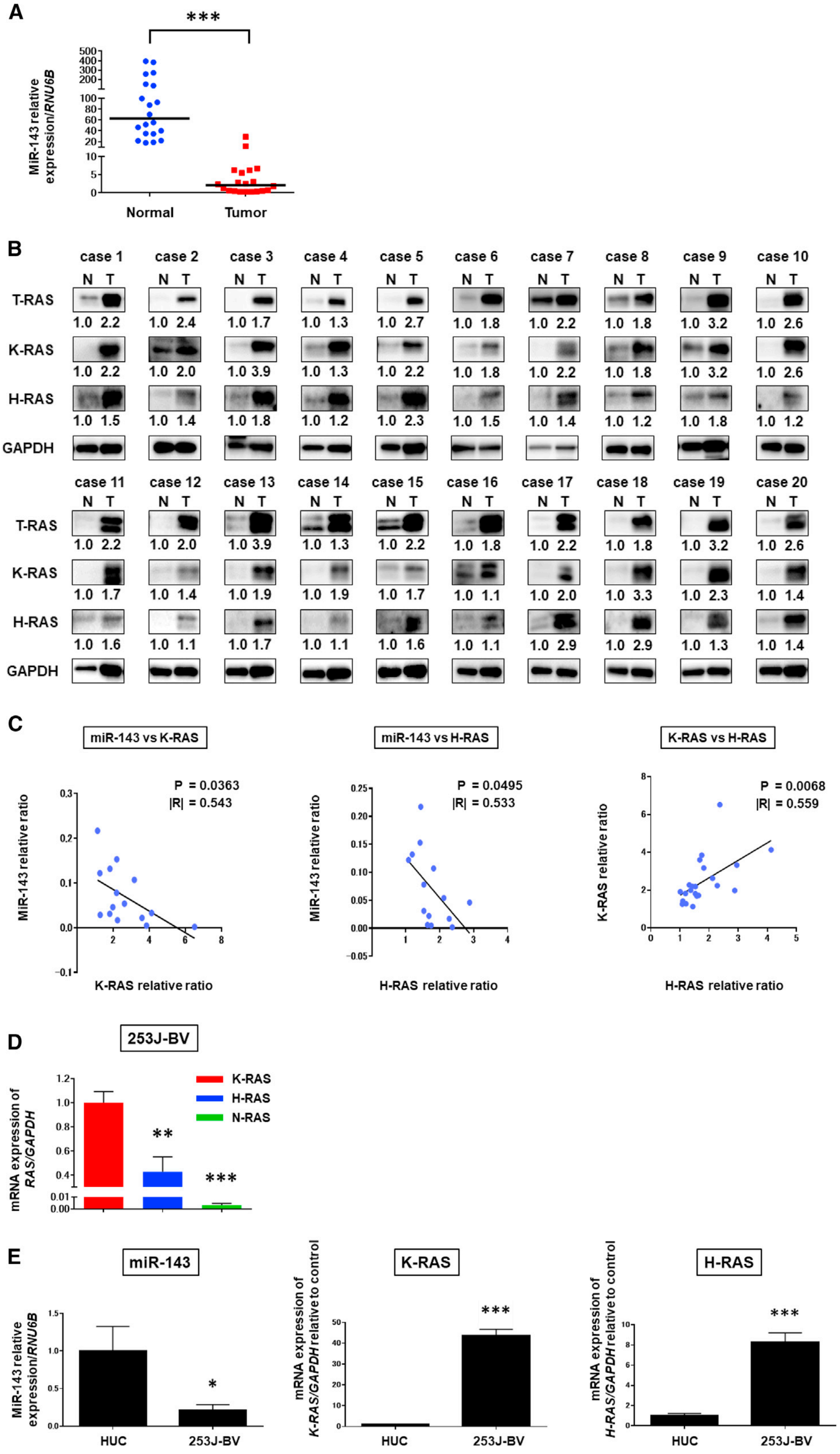

(legend on next page)

with that in HUCs (Figure 1E). To the contrary, the expression levels of K-RAS and H-RAS were upregulated in 253J-BV cells compared with those in HUCs (Figure 1E), which was remarkable in the case of K-RAS.

Synthetic miR-143s Showed a Potent Growth-Suppressive Effect on BC Cells

The structures of synthetic miR-143s (Syn-miR-143s) used in this study are shown in Figure 2A. Among them, miR-143#12 that was chemically modified with fluorine, methoxy group, phosphorylation, de-oxythymidine, and phosphorothioate in the guide strand of miR-143#1 was markedly stable in RNase-rich 10% fetal calf serum solution[20] (Figure S2A). First, we examined the growth-suppressive effect of Syn-miR-143s on 253J-BV cells to determine their anti-proliferative activity. Among them, miR-143#12 was the most effective; the half-maximum inhibitory concentration (IC_{50}) values of miR-143Am (as a standard miR-143), miR-143#1, and miR-143#12 were >40, 16.1, and 7.9 nM, respectively (Figure 2B). The protein expression levels of total RAS (T-RAS) and RAS-related Erk and Akt were downregulated in the cells transfected with either miR-143Am or Syn-miR-143s at the concentrations of IC_{50} values (Figure 2C). Also, we found that the Syn-miR-143s decreased the mRNA and protein expression levels of the target genes in a dose-dependent manner (Figure 2D). These results revealed that Syn-miR-143s worked like miR-143 and that their growth-inhibitory effects were more potent than that effect of miR-143Am. Therefore, we focused on the Syn-miR-143s in subsequent experiments.

Previously, we reported that the ectopic expression of Syn-miR-143s induced apoptosis.[17] To confirm whether apoptosis was induced in miR-143-transfected BC cells, we performed Hoechst 33342 staining of the transfected 253J-BV cells. Morphologically, the apoptotic cells estimated by the characteristic findings of apoptosis, such as nuclear fragmentation and chromatin condensation, were frequently observed, especially in miR-143#1- and #12-transfected cells, as compared with their number among the control cells (Figure 2E). Also, the levels of cleaved PARP were increased in typical miR-143#12-treated cells (Figure 2F). Next, we validated the target genes of Syn-miR-143s by using antagomiR-143. Treatment with the antagomiR-143 reversed the growth suppression of BC cells and the decrease in the levels of T-RAS, K-RAS, H-RAS, and RAS-related Erk and Akt elicited by the transfection with Syn-miR-143s (Figure 2G), which reversal was significant in the case of miR-143#12/antagomiR-143 (Figure 2H). The treatment with antagomiR-143 alone did not affect the cell growth or the expression profiles examined (data not shown). These results altogether showed that Syn-miR-143s suppressed the proliferation of 253J-BV BC cells through the decreased expression of RAS isoform proteins (K-RAS and H-RAS), Erk, and Akt by RNAi.

To confirm how Syn-miR-143s affected the expression of RAS isoform genes in the cells, we examined the expression levels of each RAS isoform mRNA after the transfection. Importantly, the levels of RAS isoform mRNAs, such as those of K-RAS, H-RAS, and N-RAS, were markedly downregulated by the transfection with Syn-miR-143s (Figure 3A). Guanosine triphosphate (GTP)-RAS activates the protein kinase Raf, which then activates MEK (MEK1 and MEK2), a mitogen-activated protein kinase (MAPK)/extracellular signal-regulated kinase (ERK) Ras-Raf-MEK-ERK in BC cells,[25] and GTP-RAS also activates the PI3K/AKT pathway, which was also proven to play a major role in bladder carcinogenesis.[26,27] To examine the activation of RAS-related-signaling pathways PI3K/AKT and MAPK/Erk, we conducted signaling inhibitor experiments in which 253J-BV cells were incubated with an AKT inhibitor or MEK inhibitor to see their effect on the proliferation of the cells. As a result, cell proliferation was significantly suppressed by either inhibitor (Figure 3B). The levels of T-RAS, K-RAS, and H-RAS proteins and the activation of PI3K/AKT- and MAPK/Erk-signaling pathways were also downregulated in the case of the AKT or MEK inhibitor (Figure 3C). Interestingly, the expression of each RAS mRNA was markedly downregulated in either AKT or MEK inhibitor-treated cells (Figure 3D), as in the case of the transfection with Syn-miR-143s (Figure 3A). These results altogether showed that the effector-signaling pathways of PI3K/AKT and Raf/MEK/Erk positively regulated the expression of RAS isoform genes, which indicated the establishment of a positive circuit for RAS expression (Figure 3E).

Next, we examined the contribution of RAS isoforms to the proliferation of 253J-BV cells by using small interfering RNAs (siRNAs) for K-RAS (siR-KRAS) and H-RAS (siR-HRAS). When 253J-BV cells were transfected with either siRNA, the ratio of cell viability was significantly decreased (Figures 4A and 4C), and the levels of RAS and RAS-related proteins were downregulated (Figures 4B and 4D). However, H-RAS protein levels were decreased even in the siR-KRAS-transfected cells (Figure 4B), whereas K-RAS protein levels were not decreased in the siR–HRAS-transfected ones (Figure 4D). Thus, both K-RAS and H-RAS would positively contribute to 253J-BV cell proliferation, but they independently affected it. Importantly, silencing of K-RAS significantly suppressed its effector-signaling molecules Akt and Erk and c-*myc* expression compared with the case of siR-HRAS.

Figure 1. Downregulated miR-143 in Clinical Bladder Cancer Samples and the Relationship between Expression of miR-143 and that of K-RAS and H-RAS in Bladder Cancer Cell Line 253J-BV

(A) Relative expression levels of miR-143 in 20 clinical bladder cancer samples. (B) Total RAS, K-RAS, and H-RAS expressions in 20 clinical bladder cancer samples as determined by western blot analysis. Densitometric values of total RAS, K-RAS, and H-RAS with respect to GAPDH of each sample were calculated, and the values are shown as each normal sample was 1.0. (C) Correlation between miR-143 and K-RAS, miR-143 and H-RAS, and K-RAS and H-RAS in clinical bladder cancer samples. (D) The mRNA expression levels of each RAS isomer in 253J-BV cells. (E) Expression levels of miR-143, K-RAS, and H-RAS in 253J-BV and normal bladder HUCs. *$p < 0.05$, **$p < 0.01$, ***$p < 0.001$.

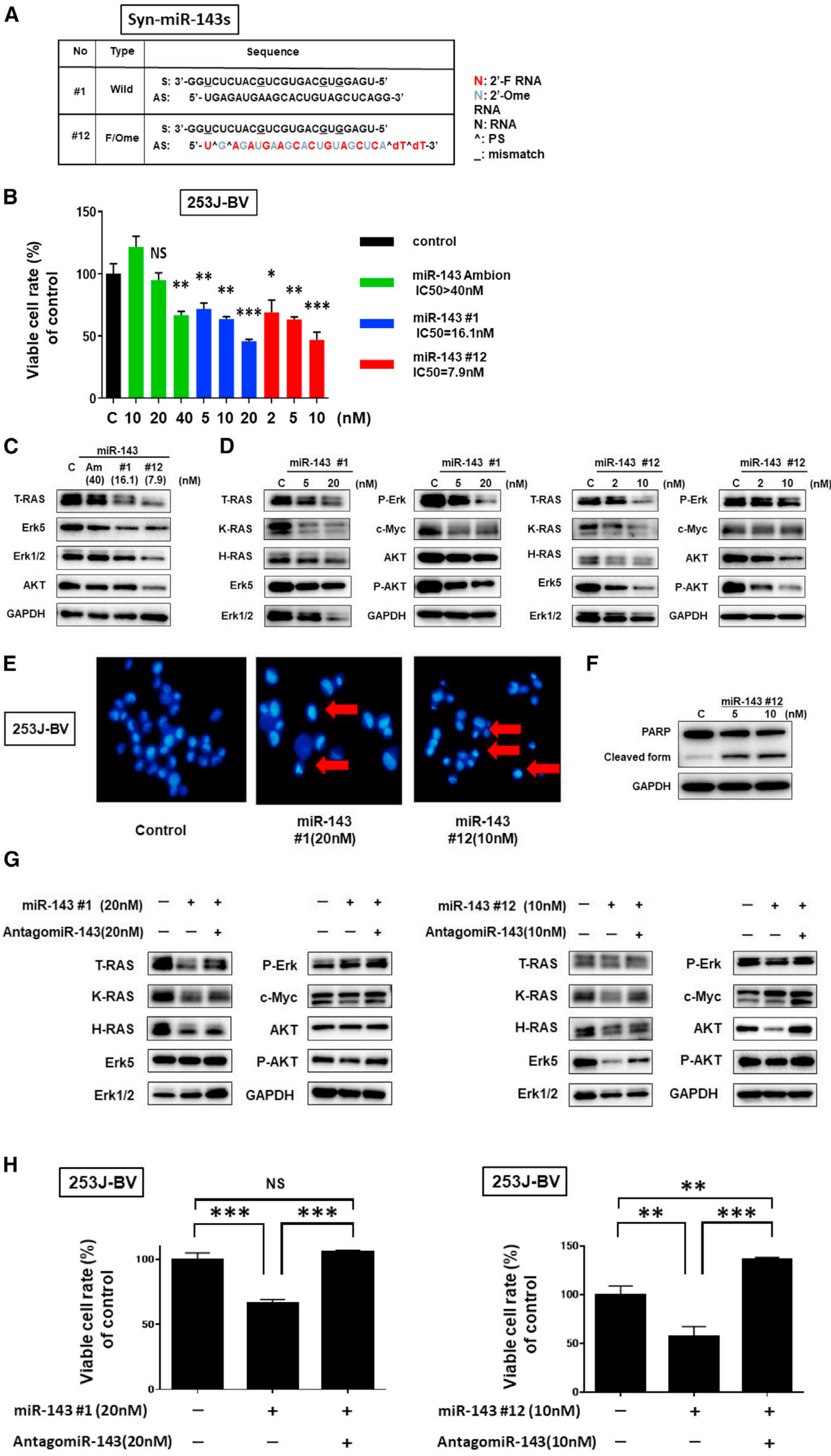

(legend on next page)

Syn-miR-143s Silences SOS1 in BC Cells

SOS1 is a guanine nucleotide exchange factor (GEF) that facilitates RAS activation by catalyzing the release of guanosine diphosphate (GDP) from RAS.[28,29] Previous reports showed a positive correlation between SOS1 high expression and several cancers, such as ovarian cancer[30] and hepatocellular carcinoma.[31] According to *in silico* prediction tool TargetScan, SOS1 has a miR-143-binding site in the 3′ UTR. Expectedly, the ectopic expression of Syn-miR-143s reduced the expression levels of SOS1; and, further, co-transfection with antagomiR-143 partially canceled the decrease elicited by each Syn-miR-143 (Figure 4E). Even when knocking down SOS1, mRNA expression levels of K-RAS, H-RAS, and N-RAS were downregulated in 253J-BV cells (Figures S3A and S3B). Also, each RAS and RAS-related proteins were decreased in these cells (Figure S3C). These results are similar to miR-143 administration (shown as in Figure 2D). When 253J-BV cells were transfected with siR-SOS1, the ratio of cell viability was significantly inhibited (Figure S3D). Western blot analysis of the clinical samples showed that, in 60% of cases tested, SOS1 expression levels were upregulated in the BC tumors compared with the normal tissue samples (Figure S3E). Also, the ratio of GTP-K-RAS:Total K-RAS was significantly decreased in a dose-dependent manner by the transfection with miR-143#12 (5 and 10 nM) (Figure 4F). Therefore, the 3′ UTR of SOS1 mRNA containing the miR-−143-binding site was cloned into the downstream of the firefly luciferase gene in a reporter plasmid for use in the luciferase reporter assay. The activity of wild-type pMIR-SOS1 was significantly reduced after the introduction of miR-143#12 into 253J-BV cells. In the case of the mutated SOS1 3′ UTR-binding site, the decrease in the luciferase activity in the wild-type was considerably abolished (Figure 4G).

Effect of miR-143/PIC Nanocarrier on Bladder Tumor Growth in Xenografted Mouse Model

Polyionic copolymer (PIC) was prepared by mixing miR-143s (aniomer) with poly (ethylene glycol)-b-poly (ornithine) (block catiomer) (Figure S4A)[32]. To evaluate the effect of Syn-miR-143s delivered by the PIC nanocarrier (Syn-miR-143s/PIC) on bladder tumor growth in a xenografted mouse model, we transplanted 253J-BV cells into the back of each mouse. We injected Syn-miR-143s/Lipo or PIC intravenously into a mouse 4 times every 72 h (Figure 5A). A significant suppression of tumor growth was observed in both groups at a low dose of total, 210 μg/kg. miR-143#1 seemed to be more potent in anti-tumor activity than did miR-143#12. The PIC group was more effective at tumor suppression than the lipofectamine group in the case of miR-143#12 (Figure 5A). No body weight loss was observed in any of the groups (Figure 5A).

To confirm the level of miR-143 in each organ, including tumor, we performed RT-PCR (Figure 5B). As a result, PIC groups showed the highest blood levels of miR-143 among the groups tested. The levels of miR-143#12 in the xenografted tumors were elevated, which would reflect the levels in the blood samples (Figure 5C). Especially, the miR-143#12/PIC group showed more accumulation of miR-143 in the tumors compared with the other groups. To understand whether miR-143 delivered by PIC inhibited tumor growth through RNAi, we performed an Argonaute2 (Ago2) loading assay (Figure 5D). The result suggested that miR-143 was included into the RNA-induced silencing complex (RISC) at least in part by binding with Ago2 protein in the cells. Also, western blot analysis of the possible target proteins of miR-143 in samples from the treated tumors gave results similar to those obtained *in vitro* (Figures 5E and 2D). The protein levels of T-RAS and RAS-related proteins were downregulated in all treated groups compared with those in the control group. Pathologically, lipofection caused hepatocyte toxicity, which was not observed in the case of PIC (Figure S4B).

Effect of Intravesical Administration of miR-143#12/PIC on Orthotopic Mouse Model

To examine the growth-inhibitory effect of miR-143#12 on the growth of bladder tumors *in vivo*, we used a mouse model bearing 253J-BV cell-xenografted tumors that was established by transplanting these cancer cells into the bladder wall of nude mice. miR-143#12/PIC, which was more effective in intravenous administration experiments, was used. The mice were treated with miR-143#12/Lipo or -143#12/PIC, and the level of miR-143 was compared in some organs (Figure S4C). Based on the results, miR-143#12 was delivered into the bladder cavity via the PIC nanocarrier, which proved to be safe and effective for general administration. As a result, miR-143#12/PIC significantly inhibited the tumor growth, when compared with that for the control-miR/PIC group, and the mice treated with miR-143#12/PIC showed almost 60% inhibition of tumor growth (Figure 6A; Figure S4D). Kaplan-Meier plots showed that the treatment group showed a significantly prolonged animal survival when compared with the control-miR/PIC group (Figure 6B). The latter group showed remarkable weight loss compared with the former one (Figure 6C). There was an inverse correlation between the bladder weight and body weight (data not shown).

We also performed the Ago2 loading assay to examine whether miR-143/PIC suppressed the tumor growth through RNAi in the tumor cells. As shown in Figure 6D, a significant amount of miR-143

Figure 2. Anti-proliferative Effects of Syn-miR-143s on 253J-BV Bladder Cancer Cells

(A) Double-stranded sequences of Syn-miR-143s (miR-143#1 and #12). (B) Cell viability at 72 h after transfection with each miR-143. Left, miR-143Am (IC_{50} > 40 nM); middle, miR-143#1 (IC_{50} = 16.1 nM); right, miR-143#12 (IC_{50} = 7.9 nM). (C) Levels of RAS and RAS-related proteins at 72 h after transfection with each miR-143, as estimated by western blot analysis. (D) Expression levels of RAS and RAS-related proteins at 72 h after transfection with miR-143#1 (5 and 20 nM) and miR-143#12 (2, 5, and 10 nM). (E) Hoechst 33342 staining showed the typical apoptotic features, such as condensed chromatin and nuclear fragmentation indicated by the red arrows, in the Syn-miR-143-treated 253J-BV cells. (F) Levels of cleaved PARP proteins at 72 h after transfection with miR-143#12 (5 and 10 nM). (G) The decrease in the levels of RAS and RAS-related proteins was reversed by co-treatment with antagomiR-143. Top, treatment with miR-143#1; bottom, treatment with miR-143#12. (H) Cell viability at 72 h after treatment with antagomiR-143 reversed the suppression of cell growth by each Syn- miR-143. Top, treatment with miR-143#1; bottom, treatment with miR-143#12.

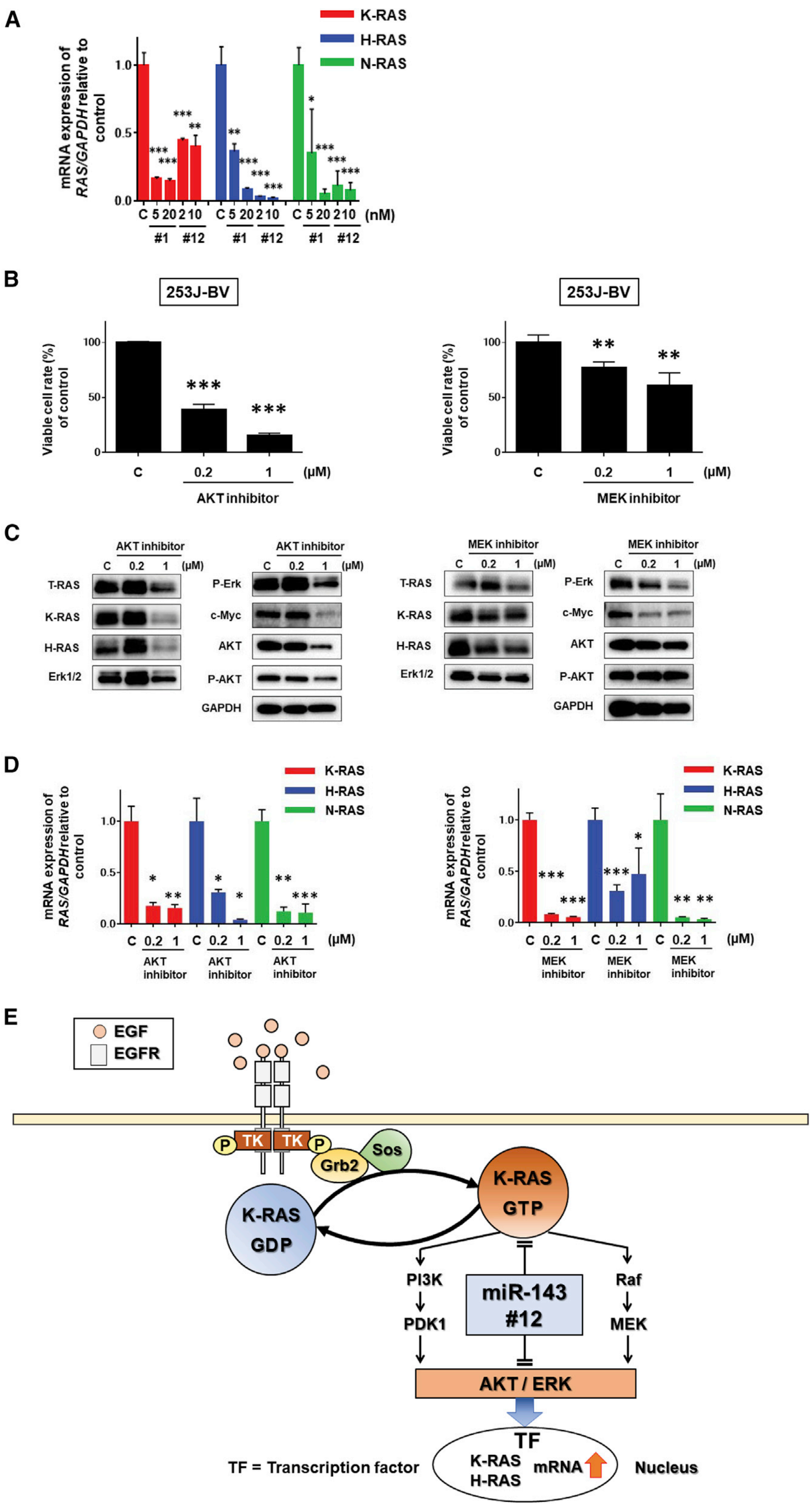

Figure 3. Effects of AKT Inhibitor or MEK Inhibitor on the Proliferation and Expression of RAS and RAS-Related Genes

(A) mRNA expression of each RAS isomer (K-RAS, H-RAS, and N-RAS) after transfection with miR-143#1 (5 and 20 nM) or miR-143#12 (2 and 10 nM). (B) Cell viability at 72 h after treatment with AKT inhibitor (0.2 and 1 μM) or MEK inhibitor (0.2 and 1 μM). (C) Levels of RAS and RAS-related proteins after treatment with AKT inhibitor (0.2 and 1 μM) or MEK inhibitor (0.2 and 1 μM). (D) mRNA expression of each RAS isomer (K-RAS, H-RAS, and N-RAS) after treatment with AKT inhibitor (0.2 and 1 μM) or MEK inhibitor (0.2 and 1 μM). (E) Schematic diagram showing the target genes of miR-143#12 and its effects on RAS networks.

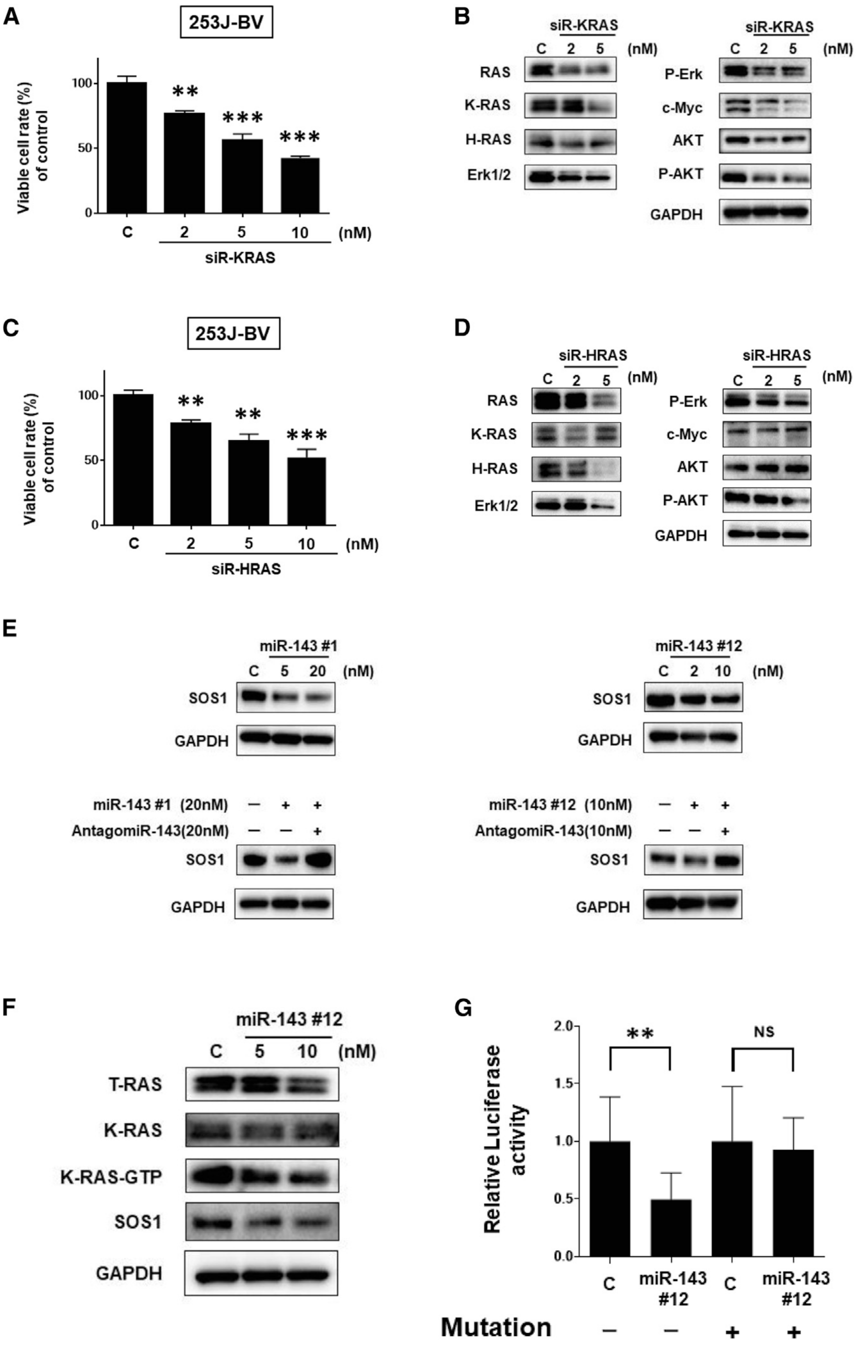

Figure 4. Effects of Silencing K-RAS and H-RAS on Cell Growth and the Ras Networks and Targeting of SOS1 by miR-143#12

(A) Cell viability at 72 h after transfection with siR-KRAS (2, 5, and 10 nM). (B) Levels of RAS and RAS-related proteins at 72 h after transfection with siR-KRAS (2 and 5 nM). (C) Cell viability at 72 h after transfection with siR-HRAS (2, 5, and 10 nM). (D) Levels of RAS and RAS-related proteins at 72 h after transfection with siR-HRAS (2 and 5 nM). (E) Use of antagomiR-143 for the validation of silencing SOS1 by miR-143#1 (left) or miR-143#12 (right). (F) Active K-RAS-GTP is downregulated by treatment with miR-143#12 through the silencing of SOS1. (G) Luciferase activities after co-transfection of 253J-BV cells with miR-143#12 and pMIR vectors having the wild-type or mutant-type miR-143-binding site in the 3′ UTR of SOS1 mRNA.

was included into RISC, as observed in the case of general injection. Western blot analysis of protein samples from the treated tumors gave results similar to those of *in vitro* experiments. Namely, the levels of RAS and RAS-related proteins in the miR-143#12/PIC group were decreased compared with those in the control-miR/PIC group, except for PI3K/AKT-signaling proteins (Figure 6E). To confirm the distributed levels of miR-143 in general organs as well as in tumors, we performed RT-PCR (Figure 6F). As expected, the treatment group of miR-143#12/PIC showed the increased levels of miR-143 mainly in the bladder tumors and blood samples. Our findings suggested that our intravesical infusion of chemically modified miR-143#12 would be a candidate of novel miRNA medicine for early BC.

DISCUSSION

In this study, we demonstrated that miR-143 was downregulated in the clinical BC samples and that there was a potent anti-cancer activity of the novel synthetic miR-143#12 toward BC cell proliferation by systemic or intravesical infusion in 253J-BV cell-xenografted mice. The synthetic miR-143#12 was selected among more than 100 kinds of miR-143 derivatives. cDNA microarray analysis indicated the similar profiles of the silenced genes after the transfection with each synthetic miR-143 used in this study (data not shown). Previously, we reported that miR-143#12 downregulated K-RAS-signaling networks through silencing K-RAS, its effector-signaling molecules Erk and Akt, and K-RAS GDP/GTP transducer SOS1.[20] In 253J-BV BC cells, both H-RAS and K-RAS are overexpressed and oncogenic. miR-143 silenced K-RAS directly and impaired the positive circuit of the RAS expression system (effector-signaling pathways of MAPK/Erk and PI3K/Akt transcription factor K-RAS and/or H-RAS).

We speculate that RTK mutations, such as those of EGFR and FGFR, would induce the overexpression of RAS in the downstream. Since miR-143#12 also silenced Akt and Erk, which inactivated growth and survival-related transcription factors, miR-143#12 could suppress the RAS-signaling networks, including the RAS-positive circuit,

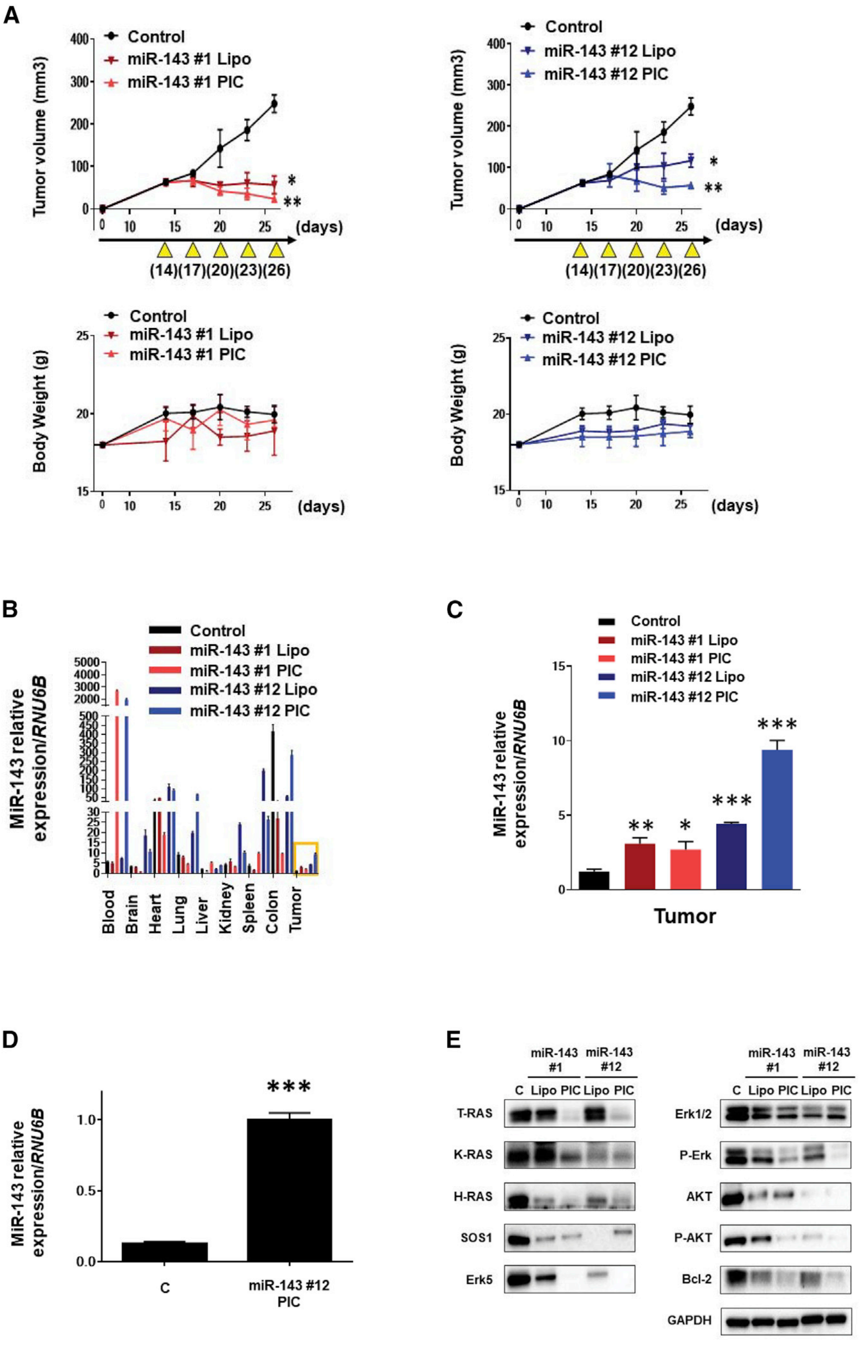

Figure 5. *In Vivo* Xenografted Mouse

(A) Comparison of the tumor volume and body weight with general administration of miR-143#1(Lipo/PIC) or miR-143#12(Lipo/PIC). (B) Tissue distribution of miR-143 in miR-143#1(Lipo or PIC) and miR-143#12(Lipo or PIC) groups. (C) Levels of miR-143 in tumor tissue samples of miR-143#1(Lipo or PIC) and miR-143#12(Lipo or PIC) groups. (D) Result of Ago2 loading assay using tumor samples from the miR-143#12/PIC group. (E) Levels of RAS and RAS-related proteins in tumor samples from miR-143#1(Lipo or PIC) and miR-143#12(Lipo or PIC) groups. A representative expression profile is shown for each group. Control-miR/PIC is indicated as "control."

to inhibit cell proliferation along with apoptosis in BC cells. On the other hand, the inhibitors for MEK and Akt suppressed the mRNA levels of both K-RAS and H-RAS, which indicated the positive circuit of their expression. K-RAS would contribute to this positive circuit rather than H-RAS, because the silencing of K-RAS, but not that of H-RAS, induced inactivation of both effector-signaling pathways. Therefore, miR-143#12, which extremely silenced K-RAS and its effector-signaling molecules Akt and Erk, as well as SOS1, could induce a potent anti-tumor effect with apoptosis in K-RAS-dominant 253J-BV BC cells, which reflected the induction of apoptosis in the case of K-RAS mutant T24 BC cells. Previously, we confirmed that ectopic expression of miR-143 induced the inhibition of cancer cell growth of BC through the regulation of cell cycle-associated genes.[33] Of course, miR-143#12 may have other anti-cancer mechanisms for BC cells.

We focused on the effect of miR-143#12 by the intravesical infusion against orthotopic BC. As a result, the significant efficacy was obtained. Before, we reported the efficacy of miR-145 by intravesical infusion.[34] In this present study, the novel synthetic miR-143#12 as well as miR-145 also exhibited a potent anti-tumor activity against BC by intravesical infusion with a PIC carrier, which was more excellent in view of the miR-143 levels in the tumor and blood samples, as well as in terms of the anti-tumor activity estimated by western blot analysis of the tumor samples. Interestingly, our result suggested that the expression levels of miR-143 were highly expressed even in brain. Therefore, miR-143#12/PIC may acquire the ability to pass the blood-brain barrier after taken by capillaries of xenografted tumor. Further investigation is required in order to qualify the ADMET (absorption, distribution, metabolism, excretion, and toxicity) of miR-143#12. Also, the finding of a potent anti-tumor effect of miR-143#12/PIC was shown in the H&E-stained tumor samples, as evidenced by severe tumor cell death with fibrosis (Figures S4B and S4D).

As to the PIC groups, we did not obtain pathologically abnormal findings in the organs such as liver and kidney in the *in vivo* experiments (Figures S4B and S4D). The cationic liposomes have been reported to be toxic to hepatocytes,[35] which were also observed in our systemic treatment. In both *in vivo* studies, we were able to confirm the effect

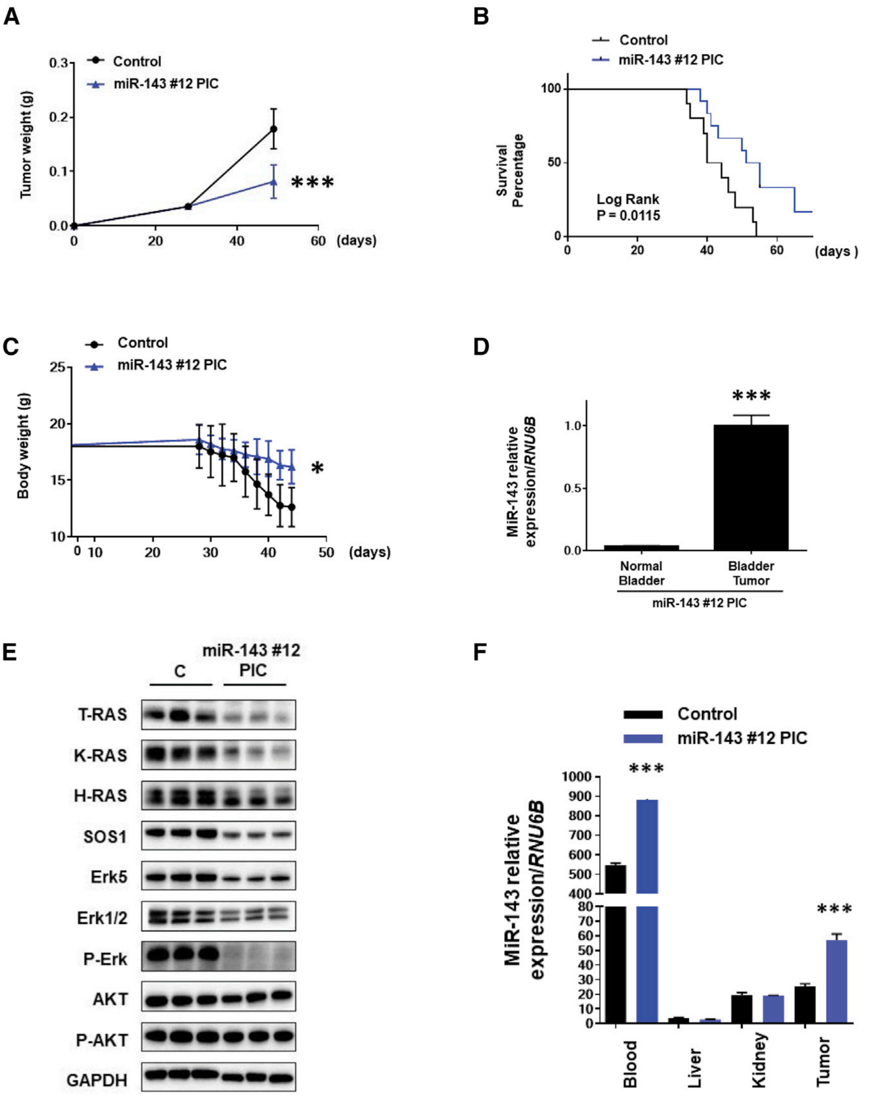

Figure 6. *In Vivo* Orthotopic Model Mouse

(A) Changes in tumor weight after treatment with control-miR/PIC or miR-143#12/PIC by intravesical injection. Approximately half of the mice were sacrificed, and the tumor weight was measured at around 30 days after the inoculation. Seven mice were assigned to each group. (B) Survival curves of miR-143#12/PIC and control-miR/PIC groups. (C) Changes in body weight of control-miR/PIC and miR-143#12/PIC groups. (D) Results of Ago2 loading assay using tumor samples from the miR-143#12/PIC group. (E) Levels of RAS and RAS-related proteins in tumor samples from the miR-143#12/PIC group. A representative protein expression profile estimated by western blot is shown. (F) Tissue distribution of miR-143 in the miR-143#12/PIC group (blood, tumor, kidney, and liver).

of miR-143#12 through RNAi, as estimated by the result of the Ago2-binding assay using samples of xenografted tumors. It is considered that the PIC nanocarrier with a hydrodynamic diameter of more than 10 nm (Figure S4A) can accumulate in the tumor, probably due to the enhanced permeability and retention (EPR) effect.[32,36] In the case of intravesical administration, we consider that PIC-conjugated miR-143 was taken into blood through the transplanted intratumoral blood vessel; however, it is not clear whether this effect worked in our mouse model based on the data of the miR-143 tissue distribution in the blood and tumor tissues (Figure 5). Further validation of the anti-cancer effect of miR-143#12, delivered by the PIC nanocarrier, on K-RAS-driven gastrointestinal cancers is now underway.

Conclusions

A novel chemically modified miR-143 systemically suppressed the RAS-signaling networks, including SOS1/RAS-, MAPK/Erk-, and PI3K/Akt-signaling pathways. In light of the therapeutic effect of systemic or intravesical administration seen *in vivo*, nucleic acid medicine using miR-143#12 would be a novel strategy for treating RAS-driven BC.

MATERIALS AND METHODS

Patients and Their Samples

All human samples were obtained from patients who had undergone biopsy or surgery for resection at Osaka Medical College Hospital (Takatsuki, Osaka, Japan). Informed consent in writing was obtained from each patient.

The consent and this study were reviewed and approved by the University Hospital Medical Information Network Center (approval R000027312), in accordance with the tenets of the Declaration of Helsinki. 20 patients with previously untreated BC or with recurrence after curative treatment were selected. The distribution according to other clinical parameters is shown in Table 1. Under a pathologist's supervision, all tissue sample pairs were collected from surgically resected tissues, with these paired samples being from the primary tumor and its adjacent non-tumor tissue in the same patient. These paired samples were examined by western blot analysis and real-time RT-PCR.

Cell Culture and Cell Viability

Human BC 253J-BV cell line was obtained from the JCRB (Japanese Collection of Research Bioresources) Cell Bank. Cell line authentication was done by short-tandem-repeat (STR) analysis, which was performed by using primers for TH01, TPOX, vWA, amelogenin, CSF1PO, D16S539, D7S820, D13S317, D5S818, and D21S11 (GenePrint 10 System; Promega, Madison, WI, USA). The cells were cultured in RPMI-1640 medium supplemented with 10% (v/v) heat-inactivated fetal bovine serum (FBS) (Sigma-Aldrich, St. Louis, MO, USA) and 2 mM L-glutamine, under an atmosphere of 95%

air and 5% CO_2 at 37°C. The number of viable cells was determined by performing the trypan blue dye exclusion test.

Transfection Experiments

253J-BV cells were seeded into 6-well plates at a concentration of 0.5×10^5/well (10%–30% confluence) on the day before the transfection. Three types of miRNA were used: the mature type of miR-143, which was a commercially available miR-143 from Ambion (mirVana miRNA mimic; Ambion, Foster City, CA, USA) (miR-143Am), and 2 types of synthetic miR-143s (Syn-miR-143s: miR-143#1 and miR-143#12). Basically, miR-143 was chemically modified in the guide strand of miR-143#1, as shown in Figure 2A. All types of miR-143s were used for the transfection of the cells, which were achieved by using cationic liposomes, Lipofectamine RNAiMAX (Invitrogen), according to the manufacturer's lipofection protocol. The nonspecific control miRNA (HSS, Hokkaido, Japan) sequence was 5′-GUA GGA GUA GUG AAA GGC C-3′, which miRNA was used as a control for nonspecific effects.[37]

In treatment experiments, the sequence of the siR-KRAS was 5′-CGG UCA UCC AGU GUU GUC AUG CAU U-3′ and that of siR-SOS1 was 5′-CGG CAU GUA CUA CAG GCC UGU UUA-3′. The effects manifested by the introduction of all types of miR-143, siR-KRAS, siR-HRAS, and siR-SOS1 into the cells were assessed at 72 h after the transfection. We used the same dose of Lipofectamine RNAiMAX in all transfection experiments.

Western Blot Analysis

Protein extraction and western blotting analysis were performed as described in previous reports.[38,39] The following primary antibodies were used: antibodies against c-Myc, p-AKT, AKT, p-ERK, ERK, PARP, and GAPDH (Cell Signaling Technology, Danvers, MA, USA); Total RAS (Abcam, Cambridge, UK); and K-RAS and H-RAS (Santa Cruz Biotechnology). Anti-rat, anti-rabbit, and horse anti-mouse immunoglobulin G (IgG) (Cell Signaling Technology) were used as secondary antibodies. GAPDH served as an internal control.

Inhibitor Experiments

253J-BV cells were seeded into 6-well plates at a concentration of 0.5×10^5/well (10%–30% confluence) on the day before the transfection. AKT inhibitor (Calbiochem, USA) and MEK inhibitor PD 98059 (Calbiochem, USA) were used for the transfection of the cells without a drug delivery system. The effects manifested by the introduction of the AKT inhibitor and MEK inhibitor into the cells were assessed at 72 h after the transfection. AntagomiR from Ambion (Anti-miR miRNA Inhibitors, Foster City, CA, USA) was used for the transfection of the cells, which was achieved by using cationic liposomes, Lipofectamine RNAi MAX (Invitrogen), according to the manufacturer's lipofection protocol.

Real-Time RT-PCR

Total RNA was isolated from cultured cells or tumor tissues by using a NucleoSpin miRNA isolation kit (TaKaRa, Otsu, Japan). RNA concentrations and purity were assessed by UV spectrophotometry. RNA integrity was checked by formaldehyde gel electrophoresis. To determine the expression levels of miR-143, we conducted qRT-PCR by using TaqMan MicroRNA Assays (Applied Biosystems) and THUNDERBIRD Probe qPCR Mix (TOYOBO,Osaka, Japan), according to the manufacturer's protocol. *RNU6B* was used as an internal control. For determination of the expression levels of *K-RAS*, *H-RAS*, *N- RAS*, *SOS1*, and *GAPDH* mRNAs, total RNA was reverse transcribed with a PrimeScript H RT reagent Kit (TaKaRa). RT-PCR was then performed with primers specific for them by using THUNDERBIRD SYBR qPCR Mix (TOYOBO). The primers for *K-RAS*, *H- RAS*, *N-RAS*, *SOS1*, and *GAPDH* were the following: *K-RAS*-sense, 5′-CCT GCT CCA TGC AGA CTG TTA-3′, and *K-RAS*-antisense, 5′-TGG GGA GAG TGA CCA TGA CT-3′; *H-RAS*-sense, 5′- TCA AAC GGG TGA AGG ACY CG-3′, and *H-RAS*-antisense, 5′-CTT CCT CCT CCT TCC GTC TG-3′; *N-RAS*-sense, 5′-GAA CCA AAC CGC AAA CGT GA-3′, and *N-RAS*-antisense, 5′-TCA AGC CCC TAT TGC TGT GG-3′; *SOS1*-sense, 5′-GGA GGA GTG TCC CAA TTT ATT AG-3′, and *SOS1*-antisense, 5′-TTT CAT TGG CTC ATG TAT AAG GG-3′; and *GAPDH*-sense, 5′-CCA CCC ATG GCA AAT TCC ATG GCA-3′, and *GAPDH*-antisense, 5′- TCT AGA CGG CAG GTC AGG TCC ACC-3′. *GAPDH* and *RNU6B* were used as internal controls. The relative expression levels were calculated by use of the ΔΔCt method.

Hoechst 33342 Staining

253J-BV cells collected at 72 h after transfection were stained with Hoechst 33342. The details of the experimental protocol were given in a previous report.[40] The apoptotic cells were observed.

Luciferase Reporter Assay

Searching the TargetScan 7.1 database (http://www.targetscan.org/) to find algorithm-based binding sites of miR-143, we found the predicted binding sites to be at positions 3,438–3,444 in the 3′ UTR of *SOS1* mRNA. The sequence region, containing the putative binding sequence of miR–143, was inserted into a pMIR-REPORT Luciferase miRNA Expression Reporter Vector (Applied Biosystems), according to the manufacturer's protocol. Moreover, we made another pMIRconstruct encompassing a mutated seed sequence for miR-143 (wild-type, CATCTCA; mutant, CAGACCA) by using a PrimeSTARH Mutagenesis Basal Kit (TaKaRa). The mutation of the vector was confirmed by sequence analysis. pRL-TK Renilla Luciferase Reporter vector (Promega, Madison WI, USA) was used as an internal control vector. 253J-BV cells were seeded into 96-well plates at a concentration of 0.4×10^4/well at 2 days before the transfection. 253J-BV cells were co-transfected with either reporter vector (0.01 mg/well each) or 20 nM miR-143#12, co-transfection of which was achieved by using Lipofectamine RNAi MAX. Luciferase activities were measured at 48 h after co-transfection by using a Dual-Glo Luciferase Assay System (Promega), according to the manufacturer's protocol. Luciferase activities were reported as the firefly luciferase:Renilla luciferase ratio.

In Vivo Xenograft Model

Animal experimental protocols were approved by the Committee for Animal Research and Welfare of Gifu University (28-87, January

31, 2017). BALB/cSLC-nu/nu (nude) mice were obtained from Japan (Hamamatsu, Japan). Human BC 253J-BV cells, which have many gene mutations, were inoculated at 2.0 × 10^6 cells/50 μL with Matrigel (Corning)/50 μL, the mixture being transplanted into the back of each mouse. The inoculation day was set as day 0. At 14 days after inoculation, we confirmed the engraftment of the tumors. miR-143s was administered in 2 formulations, except the control miRNA. In one formulation, Syn-miR-143s (#1 and #12; 250 μg/kg/1 administration) dissolved in 10 μL HEPES (1 mM) and 10 μL Opti-MEM was mixed with 1 μL Lipofectamine RNAi MAX (Invitrogen) and 80 μL saline and then injected 4 times every 72 h into a large vein. In the other formulation, Syn-miR-143s (#1 and #12; 250 μg/kg/1 administration) dissolved in 10 μL HEPES (1 mM) was mixed with 10 μL PIC comprising poly (ethylene glycol) and cationic poly (ornithine) dissolved in HEPES (25 mg/mL) and 80 μL NS (not significant) to prepare the PIC nanocarrier[32] and then generally administered 4 times, once every 72 h. The tumor volume was calculated by the following formula: 0.5236 L1 (L2),[2] where L1 is the long axis and L2 is the short axis of the tumor. Animal experiments in this study were performed in compliance with the guidelines of the Institute for Laboratory Animal Research of Gifu University and the UKCCCR Guidelines for the Welfare of Animals Used for Experimental Neoplasia. Y.A. was named in the approved experiment.

In Vivo Orthotopic Model

Human BC 253J-BV cells were implanted at 2.0 × 10^6 cells/50 μL with Matrigel (Corning)/50 μL, with the mixture being transplanted into the bladder wall of female nude mice. Once tumors had developed (average weight 0.035 g after about 28 days), they were treated with 8 intravesical injections of miR-143. miR-143 was administered in 2 ways, except the control miRNA. One way was that Syn-miR-143s (#12; 83 μg/kg/1 administration) complexed with 10 μL HEPES (1 mM) and 80 μL saline was delivered intravesically every other day. For the other administration, Syn-miR-143s (#12; 83 μg/kg/1 administration) was mixed with the block copolymer to formulate the PIC nanocarriers as described above and then delivered 8 times intravesically every other day. After the administration, some mice were sacrificed at around 30 days after the inoculation of the cells, and the tumor weight was measured. Others were observed until they died from the disease after the treatments.

Statistics

Each examination was performed in triplicate. Statistical differences between clinicopathologic parameters and the miR-143 level of tumor samples were evaluated by using Pearson's $\chi 2$ test or Fisher's extract test, unless otherwise specified. For *in vitro* and *in vivo* experiments, statistical significances of differences were evaluated by performing the two-sided Student's t test. The values were presented as the mean ± SD. A p value < 0.05 was considered to be statistically significant. The effects of intravesical injection of miR-143#12 on animal survival were evaluated by preparing Kaplan-Meier plots, with the survival duration analyzed for significance by performing log-rank survival analysis.

SUPPLEMENTAL INFORMATION

Supplemental Information can be found with this article online at https://doi.org/10.1016/j.omtm.2019.02.005.

AUTHOR CONTRIBUTIONS

Study Conception and Design, Y.Y. and Y.A.; Data Acquisition, Y.Y., T. Tsujino, K.T., and K. Heishima; Data Analysis and Interpretation, Y.Y., T. Takai, K. Minami, K.T., and Y.A.; Material and Financial Support, T.I., K. Miyata, K. Hayashi, K.K., H.A., and Y.A.; Manuscript Writing, Review, and/or Revision, Y.Y., K.T., and Y.A.; Study Supervision, H.A. and Y.A.

CONFLICTS OF INTEREST

The authors declare no conflicts of interest.

ACKNOWLEDGMENTS

This work was supported by the Project for Cancer Research and Therapeutic Evolution (P-CREATE) from Japan Agency for Medical Research and Development (AMED 16cm 0106202h0001). This work was also performed with the support of SHIONOGI. We are grateful for strong technical support by Nobuhiko Sugito, Yuki Kuranaga, Minami Kumazaki, and Haruko Shinohara (United Graduate School of Drug Discovery and Medical Information Sciences). We also thank Drs. Taizo Uchimoto, Kenkichi Saito, and Naoki Tanda (Department of Urology, Osaka Medical College) for their help with sample acquisition.

REFERENCES

1. Hall, M.C., Chang, S.S., Dalbagni, G., Pruthi, R.S., Seigne, J.D., Skinner, E.C., Wolf, J.S., Jr., and Schellhammer, P.F. (2007). Guideline for the management of nonmuscle invasive bladder cancer (stages Ta, T1, and Tis): 2007 update. J. Urol. *178*, 2314–2330.

2. Rübben, H., Lutzeyer, W., Fischer, N., Deutz, F., Lagrange, W., and Giani, G. (1988). Natural history and treatment of low and high risk superficial bladder tumors. J. Urol. *139*, 283–285.

3. Gakis, G., Efstathiou, J., Lerner, S.P., Cookson, M.S., Keegan, K.A., Guru, K.A., Shipley, W.U., Heidenreich, A., Schoenberg, M.P., Sagaloswky, A.I., et al.; International Consultation on Urologic Disease-European Association of Urology Consultation on Bladder Cancer 2012 (2013). ICUD-EAU International Consultation on Bladder Cancer 2012: Radical cystectomy and bladder preservation for muscle-invasive urothelial carcinoma of the bladder. Eur. Urol. *63*, 45–57.

4. Kim, S.P., Shah, N.D., Weight, C.J., Thompson, R.H., Wang, J.K., Karnes, R.J., Han, L.C., Ziegenfuss, J.Y., Frank, I., Tollefson, M.K., and Boorjian, S.A. (2013). Population-based trends in urinary diversion among patients undergoing radical cystectomy for bladder cancer. BJU Int. *112*, 478–484.

5. Yu, G., Yao, W., Xiao, W., Li, H., Xu, H., and Lang, B. (2014). MicroRNA-34a functions as an anti-metastatic microRNA and suppresses angiogenesis in bladder cancer by directly targeting CD44. J. Exp. Clin. Cancer Res. *33*, 779.

6. Josson, S., Chung, L.W., and Gururajan, M. (2015). microRNAs and Prostate Cancer. Adv. Exp. Med. Biol. *889*, 105–118.

7. Zhang, L., Xul, B., Chen, S., Lu, K., Liu, C., Wang, Y., Zhao, Y., Zhang, X., Liu, D., and Chen, M. (2013). The complex roles of microRNAs in the metastasis of renal cell carcinoma. J. Nanosci. Nanotechnol. *13*, 3195–3203.

8. Truta, A., Popon, T.A., Saraci, G., Ghervan, L., and Pop, I.V. (2016). Novel non invasive diagnostic strategies in bladder cancer. Clujul Med. *89*, 187–192.

9. Lin, T., Dong, W., Huang, J., Pan, Q., Fan, X., Zhang, C., and Huang, L. (2009). MicroRNA-143 as a tumor suppressor for bladder cancer. J. Urol. *181*, 1372–1380.

10. Noguchi, S., Mori, T., Hoshino, Y., Maruo, K., Yamada, N., Kitade, Y., Naoe, T., and Akao, Y. (2011). MicroRNA-143 functions as a tumor suppressor in human bladder cancer T24 cells. Cancer Lett. *307*, 211–220.
11. Cheng, T., Hu, C., Yang, H., Cao, L., and An, J. (2014). Transforming growth factor-β-induced miR-143 expression in regulation of non-small cell lung cancer cell viability and invasion capacity in vitro and in vivo. Int. J. Oncol. *45*, 1977–1988.
12. Guo, Q., Dong, B., Nan, F., Guan, D., and Zhang, Y. (2016). 5-Aminolevulinic acid photodynamic therapy in human cervical cancer via the activation of microRNA-143 and suppression of the Bcl-2/Bax signaling pathway. Mol. Med. Rep. *14*, 544–550.
13. Ahmad, I., Singh, L.B., Yang, Z.H., Kalna, G., Fleming, J., Fisher, G., Cooper, C., Cuzick, J., Berney, D.M., Møller, H., et al. (2013). Mir143 expression inversely correlates with nuclear ERK5 immunoreactivity in clinical prostate cancer. Br. J. Cancer *108*, 149–154.
14. Zhang, H., and Li, W. (2016). Dysregulation of micro-143-3p and BALBP1 contributes to the pathogenesis of the development of ovarian carcinoma. Oncol. Rep. *36*, 3605–3610.
15. Ristau, J., Staffa, J., Schrotz-King, P., Gigic, B., Makar, K.W., Hoffmeister, M., Brenner, H., Ulrich, A., Schneider, M., Ulrich, C.M., and Habermann, N. (2014). Suitability of circulating miRNAs as potential prognostic markers in colorectal cancer. Cancer Epidemiol. Biomarkers Prev. *23*, 2632–2637.
16. Shen, J.Z., Zhang, Y.Y., Fu, H.Y., Wu, D.S., and Zhou, H.R. (2014). Overexpression of microRNA-143 inhibits growth and induces apoptosis in human leukemia cells. Oncol. Rep. *31*, 2035–2042.
17. Akao, Y., Nakagawa, Y., Iio, A., and Naoe, T. (2009). Role of microRNA-143 in Fas-mediated apoptosis in human T-cell leukemia Jurkat cells. Leuk. Res. *33*, 1530–1538.
18. Pekow, J., Meckel, K., Dougherty, U., Butun, F., Mustafi, R., Lim, J., Crofton, C., Chen, X., Joseph, L., and Bissonnette, M. (2015). Tumor suppressors miR-143 and miR-145 and predicted target proteins API5, ERK5, K-RAS, and IRS-1 are differentially expressed in proximal and distal colon. Am. J. Physiol. Gastrointest. Liver Physiol. *308*, G179–G187.
19. Ngalame, N.N., Tokar, E.J., Person, R.J., Xu, Y., and Waalkes, M.P. (2014). Aberrant microRNA expression likely controls RAS oncogene activation during malignant transformation of human prostate epithelial and stem cells by arsenic. Toxicol. Sci. *138*, 268–277.
20. Akao, Y., Kumazaki, M., Shinohara, H., Sugito, N., Kuranaga, Y., Tsujino, T., Yoshikawa, Y., and Kitade, Y. (2018). Impairment of K-Ras signaling networks and increased efficacy of epidermal growth factor receptor inhibitors by a novel synthetic miR-143. Cancer Sci. *109*, 1455–1467.
21. Røtterud, R., Fosså, S.D., and Nesland, J.M. (2007). Protein networking in bladder cancer: immunoreactivity for FGFR3, EGFR, ERBB2, KAI1, PTEN, and RAS in normal and malignant urothelium. Histol. Histopathol. *22*, 349–363.
22. Ouerhani, S., and Elgaaied, A.B. (2011). The mutational spectrum of HRAS, KRAS, NRAS and FGFR3 genes in bladder cancer. Cancer Biomark. *10*, 259–266.
23. Yeh, C.W., Huang, S.S., Lee, R.P., and Yung, B.Y. (2006). Ras-dependent recruitment of c-Myc for transcriptional activation of nucleophosmin/B23 in highly malignant U1 bladder cancer cells. Mol. Pharmacol. *70*, 1443–1453.
24. Chen, X., Guo, X., Zhang, H., Xiang, Y., Chen, J., Yin, Y., Cai, X., Wang, K., Wang, G., Ba, Y., et al. (2009). Role of miR-143 targeting KRAS in colorectal tumorigenesis. Oncogene *28*, 1385–1392.
25. Juanpere, N., Agell, L., Lorenzo, M., de Muga, S., López-Vilaró, L., Murillo, R., Mojal, S., Serrano, S., Lorente, J.A., Lloreta, J., and Hernández, S. (2012). Mutations in FGFR3 and PIK3CA, singly or combined with RAS and AKT1, are associated with AKT but not with MAPK pathway activation in urothelial bladder cancer. Hum. Pathol. *43*, 1573–1582.
26. Cirone, P., Andresen, C.J., Eswaraka, J.R., Lappin, P.B., and Bagi, C.M. (2014). Patient-derived xenografts reveal limits to PI3K/mTOR- and MEK-mediated inhibition of bladder cancer. Cancer Chemother. Pharmacol. *73*, 525–538.
27. Calderaro, J., Rebouissou, S., de Koning, L., Masmoudi, A., Hérault, A., Dubois, T., Maille, P., Soyeux, P., Sibony, M., de la Taille, A., et al. (2014). PI3K/AKT pathway activation in bladder carcinogenesis. Int. J. Cancer *134*, 1776–1784.
28. Ilinskaya, O.N., Singh, I., Dudkina, E., Ulyanova, V., Kayumov, A., and Barreto, G. (2016). Direct inhibition of oncogenic KRAS by Bacillus pumilus ribonuclease (binase). Biochim. Biophys. Acta *1863* (7 Pt A), 1559–1567.
29. Egan, S.E., Giddings, B.W., Brooks, M.W., Buday, L., Sizeland, A.M., and Weinberg, R.A. (1993). Association of Sos Ras exchange protein with Grb2 is implicated in tyrosine kinase signal transduction and transformation. Nature *363*, 45–51.
30. Xiao, Z.H., Linghu, H., and Liu, Q.F. (2016). [Expressions of Ras and Sos1 in epithelial ovarian cancer tissues and their clinical significance]. Nan Fang Yi Ke Da Xue Xue Bao *36*, 1502–1507.
31. Liu, K., Jiang, T., Ouyang, Y., Shi, Y., Zang, Y., Li, N., Lu, S., and Chen, D. (2015). Nuclear EGFR impairs ASPP2-p53 complex-induced apoptosis by inducing SOS1 expression in hepatocellular carcinoma. Oncotarget *6*, 16507–16516.
32. Hayashi, K., Chaya, H., Fukushima, S., Watanabe, S., Takemoto, H., Osada, K., Nishiyama, N., Miyata, K., and Kataoka, K. (2016). Influence of RNA Strand Rigidity on Polyion Complex Formation with Block Catiomers. Macromol. Rapid Commun. *37*, 486–493.
33. Noguchi, S., Yasui, Y., Iwasaki, J., Kumazaki, M., Yamada, N., Naito, S., and Akao, Y. (2013). Replacement treatment with microRNA-143 and -145 induces synergistic inhibition of the growth of human bladder cancer cells by regulating PI3K/Akt and MAPK signaling pathways. Cancer Lett. *328*, 353–361.
34. Inamoto, T., Taniguchi, K., Takahara, K., Iwatsuki, A., Takai, T., Komura, K., Yoshikawa, Y., Uchimoto, T., Saito, K., Tanda, N., et al. (2015). Intravesical administration of exogenous microRNA-145 as a therapy for mouse orthotopic human bladder cancer xenograft. Oncotarget *6*, 21628–21635.
35. Roursgaard, M., Knudsen, K.B., Northeved, H., Persson, M., Christensen, T., Kumar, P.E.K., Permin, A., Andresen, T.L., Gjetting, T., Lykkesfeldt, J., et al. (2016). In vitro toxicity of cationic micelles and liposomes in cultured human hepatocyte (HepG2) and lung epithelial (A549) cell lines. Toxicol. In Vitro *36*, 164–171.
36. Matsumura, Y., and Maeda, H. (1986). A new concept for macromolecular therapeutics in cancer chemotherapy: mechanism of tumoritropic accumulation of proteins and the antitumor agent smancs. Cancer Res. *46*, 6387–6392.
37. Akao, Y., Nakagawa, Y., Hirata, I., Iio, A., Itoh, T., Kojima, K., Nakashima, R., Kitade, Y., and Naoe, T. (2010). Role of anti-oncomirs miR-143 and -145 in human colorectal tumors. Cancer Gene Ther. *17*, 398–408.
38. Noguchi, S., Iwasaki, J., Kumazaki, M., Mori, T., Maruo, K., Sakai, H., Yamada, N., Shimada, K., Naoe, T., Kitade, Y., and Akao, Y. (2013). Chemically modified synthetic microRNA-205 inhibits the growth of melanoma cells in vitro and in vivo. Mol. Ther. *21*, 1204–1211.
39. Yamada, N., Tsujimura, N., Kumazaki, M., Shinohara, H., Taniguchi, K., Nakagawa, Y., Naoe, T., and Akao, Y. (2014). Colorectal cancer cell-derived microvesicles containing microRNA-1246 promote angiogenesis by activating Smad 1/5/8 signaling elicited by PML down-regulation in endothelial cells. Biochim. Biophys. Acta *1839*, 1256–1272.
40. Taniguchi, K., Sugito, N., Kumazaki, M., Shinohara, H., Yamada, N., Nakagawa, Y., Ito, Y., Otsuki, Y., Uno, B., Uchiyama, K., and Akao, Y. (2015). MicroRNA-124 inhibits cancer cell growth through PTB1/PKM1/PKM2 feedback cascade in colorectal cancer. Cancer Lett. *363*, 17–27.

OMTM, Volume *13*

Supplemental Information

Anti-cancer Effects of a Chemically Modified miR-143 on Bladder Cancer by Either Systemic or Intravesical Treatment

Yuki Yoshikawa, Kohei Taniguchi, Takuya Tsujino, Kazuki Heishima, Teruo Inamoto, Tomoaki Takai, Koichiro Minami, Haruhito Azuma, Kanjiro Miyata, Kotaro Hayashi, Kazunori Kataoka, and Yukihiro Akao

Materials and methods

Clinical data analysis

Clinical sample data was quoted from available database c-Bioportal for cancer genomics (http://www.cbioportal.org/). Clinical samples included from the combination of 9 cohorts are 1412 specimens. K-RAS, H-RAS and N-RAS mutation in the sample were analyzed.

RNA degradation

In order to confirm the resistance of chemically modified miR-143 to RNAse, RNA degradation experiment was carried out. Compared Ambion's miR-143 and chemically modified miR-143s. MiR-143s are administered to 10% FBS solution media and RNA equilibrium is established by injecting PCI solution in 1, 2, 3, 4, 5, 10, 20 and 30 minutes. RNA was extracted from the solution and the amount of miR143 was measured by real time PCR. The ratio of phenol, chloroform, and isoamyl alcohol are 25: 24: 1.

Patients and their samples

All human samples were obtained from patients who had undergone biopsy or surgery for resection at Osaka Medical College Hospital (Takatsuki, Osaka, Japan). Informed consent in writing was obtained from each patient. This is exactly the same as the sample used in Fig. 1.

Transfection experiments

253J-BV cells were seeded into 6-well plates at a concentration of 0.5 × 10^5 per well (10-30% confluence) on the day before the transfection. siRNAs were used: In treatment experiments, the sequence of the siR-SOS1, 5'-CGG CAU GUA CUA CAG GCC UGU UUA-3'. The effects manifested by the introduction of siR-SOS1 into the cells were assessed at 72 h after the transfection as mRNA expression level, protein levels of RAS and RAS related genes, and cell proliferation. We used the same dose of Lipofectamine^{TM} RNAiMAX in all transfection experiments.

Real-time reverse transcription PCR

The detail methods of isolation of RNA and RT-PCR were described in main document. The primers for *SOS1* and *GAPDH* were the following: *SOS1*-sense, 5'-GGA GGA GTG TCC CAA TTT ATT AG-3', and *SOS1*-antisense, 5'-TTT CAT TGG CTC ATG TAT AAG GG-3'; *GAPDH*-sense, 5'-CCA CCC ATG GCA AAT TCC ATG GCA-3', and *GAPDH*-antisense, 5'-TCT AGA CGG CAG GTC AGG TCC ACC-3'.*GAPDH* was used as internal controls. The relative expression levels were calculated by use of the ΔΔCt method.

Western blot analysis

Protein extraction and Western blotting analysis were performed as described in main document. The following primary antibodies were used:

antibodies against, c-Myc, p-AKT, AKT, p-ERK, ERK, SOS1, and GAPDH (Cell Signaling Technology, Inc., Danvers, MA, USA);, Total RAS (Abcam, Cambridge, UK);, and K-RAS, H-RAS (Santa Cruz Biotechnology). Anti-rat, anti-rabbit, and horse anti-mouse IgG (Cell Signaling Technology) were used as secondary antibodies. GAPDH served as an internal control.

In vivo orthotopic model

The method of transplantation of the tumor and time table of administering the medicine were carried out as described in the main document. MiR-143 was administered in 2 ways except the control miRNA. One way was that Syn-miR-143s (#12; 83 μg/kg per 1 administration) complexed with 10 μl of HEPES (1mM), 10 μl of Opti-MEM that had been incubated with 1 μl of Lipofectamine^{TM} RNAiMAX (Invitrogen), and 80 μl of saline were delivered intravesically every other day. For the other administration, Syn-miR-143s (#12; 83 μg/kg per 1 administration) was mixed with the block copolymer to formulate the PIC nanocarriers as described above and then delivered 8 times intravesically every other day. After the administration, some mice were sacrificed at around 30 days after the inoculation of the cells. RNA was extracted from several organs. Some organs were stained with hematoxylin and eosin (H-E).

Supplementary Figure.1

A

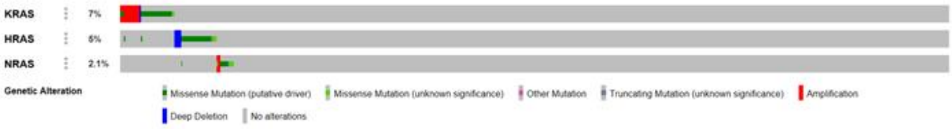

Supplementary Figure.2

A

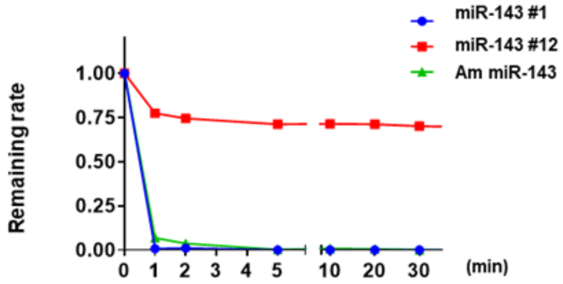

Supplementary Figure.3

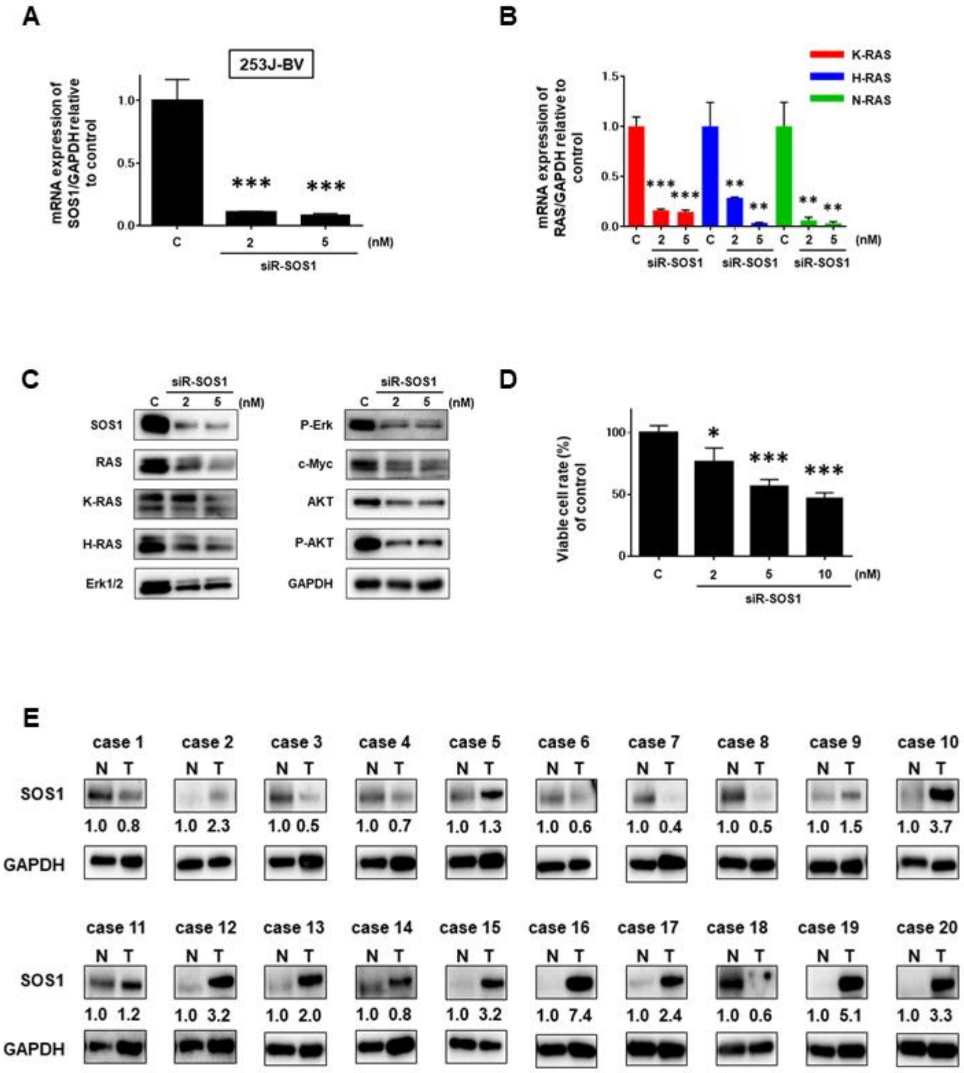

Supplementary Figure.4

A

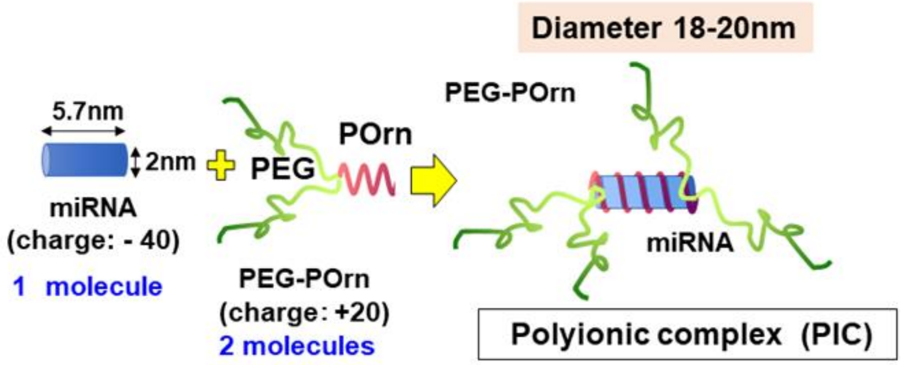

B

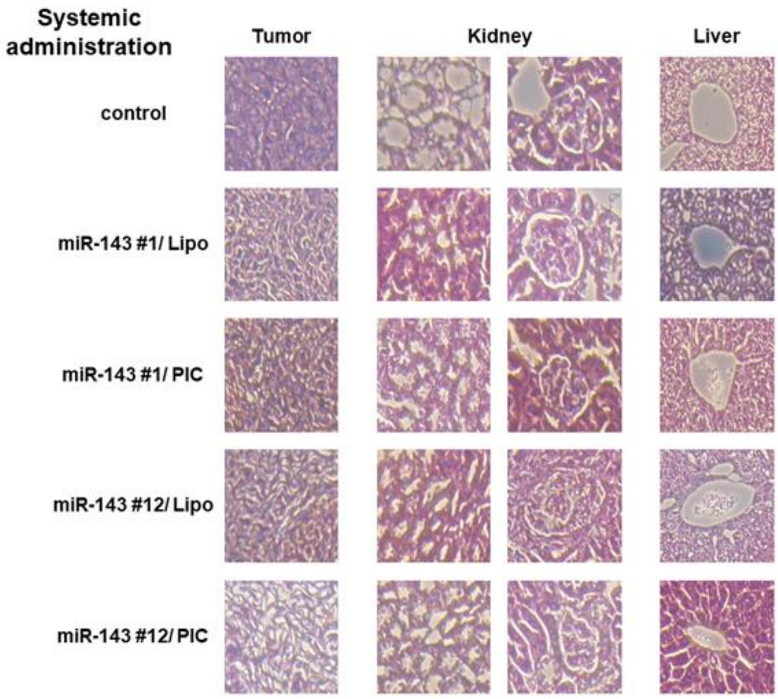

C

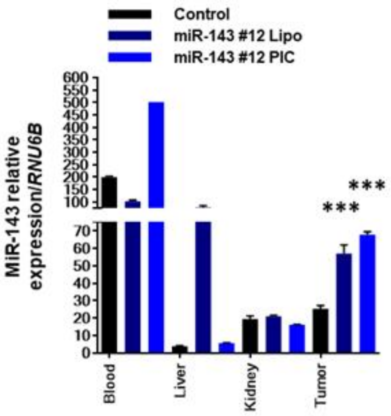

D

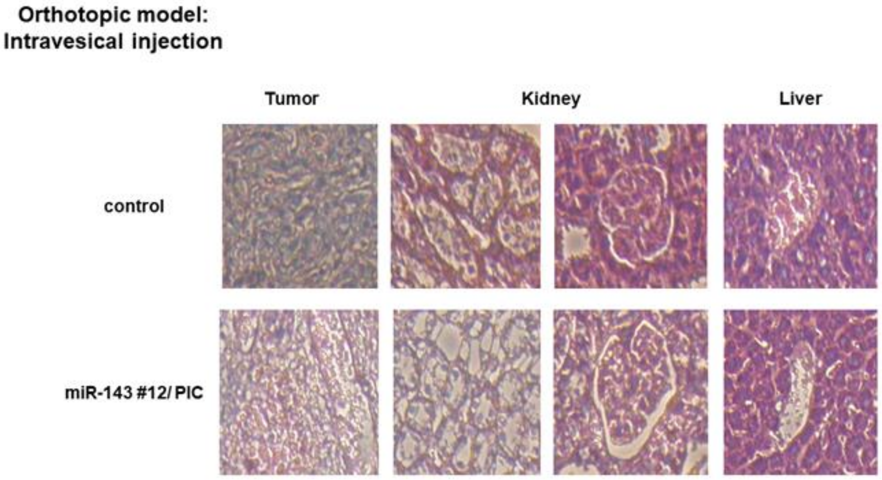
Orthotopic model:
Intravesical injection
Tumor
Kidney
Liver
control
miR-143 #12/ PIC

Supplemental Figures

Supplementary Figure 1. Mutation of RAS in clinical samples of bladder cancer.

A. Clinical significance of RAS mutation in BC. Patients are in 9 studies from available datasets. cBioportal for cancer genomics (http://www.cbioportal.org/) was used.

Supplementary Figure 2. Stability of miR-143s used in this study

A. Decay of miR-143 after the incubation of miR-143s in 5%FCS solution.

Supplementary Figure 3. Role of SOS1 in regulating RAS gene in BC cells.

A. mRNA expression of SOS1 after transfection with siR-SOS1 (2 nM, 5 nM).

B. mRNA expression of RAS isomer (K-RAS, H-RAS, and N-RAS) at 72 h after transfection with siR-SOS1 (2 nM, 5 nM).

C. Levels of RAS and RAS-related proteins at 72 h after transfection with siR-SOS1 (2 nM, 5 nM).

D. Cell viability at 72 h after transfection with siR-SOS1 (2 nM, 5 nM, 10 nM).

E. SOS1 expression in 20 clinical bladder cancer samples as determined

by Western blot analysis.

Supplementary Figure 4. Formulation of PIC and histopathological findings for tumor, kidney, and liver after treatments of 253J-BV cell-xenografted mice

A. Formulation of each miR-143/PIC. POrn: poly(Ornithine)

B. Histopathology of tumor/kidney/liver after treatment of subcutaneous xenograft mice with miR-143#1(Lipo or PIC) or miR-143#12(Lipo or PIC).

C. Tissue distribution of miR-143 in the miR-143#12/Lipo and miR-143#12/PIC groups (Blood/Tumor/ Kidney/ Liver).

D. Histopathology of tumor/kidney/liver after intravesical infusion with miR-143#12/PIC in orthotopic xenograft model.
